# High Resolution HLA ∼A, ∼B, ∼C, ∼DRB1, ∼DQA1, and ∼DQB1 Diversity in South African Populations

**DOI:** 10.3389/fgene.2022.711944

**Published:** 2022-03-04

**Authors:** Mqondisi Tshabalala, Juanita Mellet, Kuben Vather, Derrick Nelson, Fathima Mohamed, Alan Christoffels, Michael S. Pepper

**Affiliations:** ^1^ Department of Immunology, Institute for Cellular and Molecular Medicine, Faculty of Health Sciences, University of Pretoria, Pretoria, South Africa; ^2^ South African Medical Research Council (SAMRC) Extramural Unit for Stem Cell Research and Therapy, Faculty of Health Sciences, University of Pretoria, Pretoria, South Africa; ^3^ South African National Blood Service (SANBS), Roodepoort, South Africa; ^4^ SAMRC Bioinformatics Unit, South African National Bioinformatics Institute, University of the Western Cape, Bellville, South Africa

**Keywords:** high resolution typing, HLA diversity, South Africa, haplotype frequencies, allele frequencies, human leukocyte antigen (HLA)

## Abstract

**Background:** Lack of HLA data in southern African populations hampers disease association studies and our understanding of genetic diversity in these populations. We aimed to determine HLA diversity in South African populations using high resolution HLA ∼A, ∼B, ∼C, ∼DRB1, ∼DQA1 and ∼DQB1 data, from 3005 previously typed individuals.

**Methods:** We determined allele and haplotype frequencies, deviations from Hardy-Weinberg equilibrium (HWE), linkage disequilibrium (LD) and neutrality test. South African HLA class I data was additionally compared to other global populations using non-metrical multidimensional scaling (NMDS), genetic distances and principal component analysis (PCA).

**Results:** All loci strongly (*p* < 0.0001) deviated from HWE, coupled with excessive heterozygosity in most loci. Two of the three most frequent alleles, HLA ∼DQA1*05:02 (0.2584) and HLA ∼C*17:01 (0.1488) were previously reported in South African populations at lower frequencies. NMDS showed genetic distinctness of South African populations. Phylogenetic analysis and PCA clustered our current dataset with previous South African studies. Additionally, South Africans seem to be related to other sub-Saharan populations using HLA class I allele frequencies.

**Discussion and Conclusion:** Despite the retrospective nature of the study, data missingness, the imbalance of sample sizes for each locus and haplotype pairs, and induced methodological difficulties, this study provides a unique and large HLA dataset of South Africans, which might be a useful resource to support anthropological studies, disease association studies, population based vaccine development and donor recruitment programs. We additionally provide simulated high resolution HLA class I data to augment the mixed resolution typing results generated from this study.

## Introduction

The human leukocyte antigen (HLA) gene region is considered to be one of the most polymorphic regions in the human genome (Mungall et al., 2003; Wong et al., 2013). Currently, there are 30 862 HLA alleles listed (https://www.ebi.ac.uk/ipd/imgt/hla/stats.html) in the IMGT/HLA database (3.45.0 release of July 2021). HLA genes encode proteins involved in antigen presentation (Robinson et al., 2013), and play a key determining role in transplantation clinical outcomes (Beatty et al., 2000; Carrington and O'Brien, 2003; Ndung’u et al., 2005; Brander et al., 2006; Ovsyannikova and Poland, 2011; Chen et al., 2012; Ramsay, 2012; Garamszegi, 2014). Despite the growing documented evidence of genetic diversity in Africans (Chen et al., 1995; Zietkiewicz et al., 1997; Jorde et al., 2000; Prugnolle et al., 2005; Disotell, 2012), there remains an information gap on HLA diversity in these populations (reviewed in Tshabalala et al. (2015)). This lack of HLA data hampers disease association studies (reviewed in Dyer et al. (2013), population-specific vaccine development (Gourraud et al., 2015) and programs aimed at donor recruitment into registries (Edinur et al., 2016). Additionally, there is a high disease burden in these populations (WHO, 2013). Understanding HLA diversity will compliment efforts to eliminate these health challenges.

In addition to its key role in the human immune system, HLA has been used to understand human genetic diversity, population genetics and anthropology. HLA has been widely used to understand genetic relatedness of different populations as well as demographic events in those populations (Sanchez-Mazas and Meyer, 2014). The HLA genetic makeup of populations provides insight into their histories including selective pressures by pathogens (Prugnolle et al., 2005), migration, admixture and changes in population size (Parham and Ohta, 1996; Kijak et al., 2009; Buhler and Sanchez-Mazas, 2011; Sanchez-Mazas et al., 2011). The availability of population HLA data is thus critical to understanding peopling history and general evolution of the human immune system (Burrell and Disotell, 2009; Meyer et al., 2018).

The South African population comprises 59.6 million people (Statistics South Africa, 2022). The presence of a high disease burden in the population is one factor which may drive high genetic diversity, including HLA diversity. Additionally, a large proportion of the population harbors residual DNA sequences from *Homo naledi* (Berger et al., 2015), one of the oldest Hominid ancestors, allowing accumulation of polymorphisms over thousands of years. Additionally, new and low frequency HLA alleles have been reported in South African populations (Paximadis et al., 2012; Hayhurst et al., 2015) supporting the idea of high genetic diversity in these populations (May et al., 2013; Choudhury et al., 2017). We previously described allele and haplotype frequencies from the South African Bone Marrow Registry (SABMR) (Tshabalala et al., 2018) in an effort to understand HLA diversity in South Africans. The current study is aimed at improving our understanding of HLA diversity in South Africans using retrospectively typed individuals in the National Health Laboratory Services (NHLS) and the South African National Blood Services (SANBS). We additionally sought to compare HLA data from South Africans with other global populations using population genetics approaches.

## Materials and Methods

### Study Population, Human Leukocyte Antigen Data Access and Ethics

Approval for this study was granted by the Research Ethics Committee of the University of Pretoria, Faculty of Health Sciences (approval no. 220/2015), the SANBS Human Research Ethics Committee (SANBS HREC) and NHLS Academic Affairs and Research. We analyzed a combined total of 3005 high resolution (four digit typing HLA ∼A, HLA ∼B, HLA ∼C, HLA ∼DRB1, HLA ∼DQA1 and HLA ∼DQB1) results from the SANBS and the NHLS. The retrospective high resolution typing dataset (defined in the context of this study as four digit typing resolution) has been assembled from higher resolution DNA based methods in the SANBS and the NHLS. All available HLA data from the SANBS (up to 20 November 2016) plus the NHLS data (05 June 2003 to 12 April 2016) was accessed. The NHLS offers national diagnostic pathology services (http://www.nhls.ac.za/) whilst the SANBS aims to supply safe blood and blood products to the local population (https://sanbs.org.za/). Only HLA data was accessed; no additional data was accessed due to ethical considerations. Participants’ personal identifiers were not accessed to maintain confidentiality following the Helsinki ethical guidelines (Association, 2013). All the accessed HLA data was checked for allele validity, and all pre-2010 nomenclature designations were converted using current nomenclature conversion tables and conversion tools provided by IMGT/HLA (https://www.ebi.ac.uk) based on the IMGT/HLA database (3.45.0 release of July 2021) (https://www.ebi.ac.uk/ipd/imgt/hla/stats.html). HLA data missingness in our dataset is defined as the lack of typing methods to call two alleles at a given locus, resulting in one allele for that individual at that particular locus. Unfortunately, a distinction between homozygous typing and data missingness could not be established due to the retrospective nature of the study.

### Statistical Analysis

High (four digit) resolution data was analyzed to estimate linkage disequilibrium (LD), Hardy-Weinberg equilibrium (HWE) proportions, homozygosity test of neutrality, and allele and haplotype frequencies. Allele and haplotype frequencies were estimated by resolving phase and allelic ambiguities using the expectation-maximization (EM) algorithm (Excoffier and Slatkin, 1995; Eberhard et al., 2013) both implemented in Python for population genomics (PyPop) version 0.7.0 (Lancaster et al., 2007) and gene [RATE] tools (https://hla-net.eu/tools/basic-statistics/) (Nunes, 2016). Excoffier and Slatkin (1995) allows estimation of random haplotypes based on sample allele frequencies. For pairwise LD, we used Hedrick’s D′ (Hedrick, 1987) and Cramer’s V Statistic (W_n_) (Cramér, 1946), all implemented in PyPop version 0.7.0 (Lancaster et al., 2007). HLA genotypes were converted to Arlequin version 3.5.2 (Lancaster et al., 2007) input files using CREATE version 1.37 software (Coombs et al., 2008) to assess deviations from HWE [modified hidden Markov chain (Guo and Thompson, 1992) with 100 000 dememorization steps]. Slatkin’s implementation of Ewens-Watterson homozygosity test of neutrality (Slatkin, 1994; 1996) was done in PyPop version 0.7.0 (Lancaster et al., 2007). In addition to allele frequencies, cumulative allele frequencies from the South African population were plotted for high resolution typing data sets.

### Population Comparison

To better understand HLA diversity in our dataset, we compared our findings to other global populations. Our data was compared with multiple population datasets from gene [RATE] tools (Nunes, 2016) defined world regions by non-metrical multidimensional scaling (NMDS) analysis. Due to the HLA mixed resolution typing nature and data missingness in our dataset, we performed HLA ∼A, ∼B and ∼C (HLA class I) completion of our data set to get high resolution (four digit typing) data using the PhyloD tool as previously described (Listgarten et al., 2008). The PhyloD HLA completion tool uses statistical *in silico* methods to probabilistically predict four digit HLA class I alleles (Listgarten et al., 2008). We further compared our class I HLA allele frequency data with PhyloD generated allele frequency data (Listgarten et al., 2008), and 28 other publicly available HLA ∼A, ∼B and ∼C allele frequencies (four digit resolution) as well as sub-Saharan African data from the Allele Frequencies Net Database (AFND) (González-Galarza et al., 2015) including previous South African studies (Loubser et al., 2017a; b;Grifoni et al., 2018; Tshabalala et al., 2018). Specifically, our HLA data (RSA) was compared with the following AFND defined populations (population codes we used for phylogenetic analysis): Burkina Faso Fulani (BFF) (Modiano et al., 2001), Burkina Faso Mossi (BFM) (Modiano et al., 2001), Burkina Faso Rimaibe (BFR) (Modiano et al., 2001), Cameroon Baka Pygmy (CBP) (Torimiro et al., 2006), Cameroon Bakola Pygmy (CBkP) (Bruges Armas et al., 2003), Cameroon Bamileke (CaB) (Torimiro et al., 2006), Cameroon Beti (CBt) (Torimiro et al., 2006), Cameroon Sawa (CSw) (Torimiro et al., 2006), Central African Republic Mbenzele Pygmy (CARMP) (Bruges Armas et al., 2003), Ghana Ga-Adangbe (GGA) (Norman et al., 2013), Kenya (KEN) (Luo et al., 2002), Kenya Luo (KENL) (Cao et al., 2004), Kenya Nandi (KENN) (Cao et al., 2004), Kenya, Nyanza Province, Luo tribe (KENNy) (Arlehamn et al., 2017), PhyloD generated data (PSA) (Listgarten et al., 2008), Rwanda (RWA) (Tang et al., 2000), Senegal Niokholo Mandenka (SenMAND) (Sanchez-Mazas et al., 2000), South Africa Black (SoAB) (Paximadis et al., 2012), South Africa Caucasians (SoAC) (Paximadis et al., 2012), South Africa Natal Tamil (SANT) (Hammond and Anley, 2006), South Africa Natal Zulu (SANZ) (Hammond et al., 2006), South Africa Worcester (WOR) (Grifoni et al., 2018), South African Bone Marrow Registry (SAB) (Tshabalala et al., 2018), South African Indian population (SAI) (Loubser et al., 2017a), South African Mixed ancestry (RMX) (Loubser et al., 2017b), Uganda Kampala (UgaKam) (Cao et al., 2004), Uganda Kampala pop 2 (UgaKam2) (Kijak et al., 2009), Zambia Lusaka (ZaL) (Cao et al., 2004) and Zimbabwe Harare Shona (ZiHS) (Louie et al., 2006). HLA class I allele frequencies from the above 30 populations were used to compute pairwise population differentiation (F_ST_) and Nei’s genetic distances (Nei, 1972) in POPTREE software (Takezaki et al., 2010; 2014). An unrooted tree was constructed based on the Neighbour-Joining (NJ) method (Saitou and Nei, 1987) implemented in POPTREE software (Takezaki et al., 2010; 2014) using Nei’s genetic distances. The pairwise F_ST_ matrix was used for PCA in ClustVis (a web tool for visualizing clustering of multivariate data using PCA and heat map) (Metsalu and Vilo, 2015). Additionally, the South African HLA ∼A, ∼B and ∼C cumulative allele frequencies (four digit resolution) generated in this study were compared to Kenyan, Ugandan and Zambian cumulative frequencies from the AFND (González-Galarza et al., 2015). All HLA alleles were sorted in descending order according to their frequencies, and cumulative frequencies were plotted according to the total number of alleles at a particular locus.

## Results

### HWE Proportions and Neutrality Test

All loci showed a strong significant deviation from the expected HWE proportions (*p* < 0.0001) as detailed in Table 1. The Ewens-Watterson neutrality test showed negative and significant F*nd* values for HLA ∼A (*p* < 0.0001) and ∼DQB1 (*p* = 0.0133) (Table 2). This indicates homozygosity which is suggestive of balancing selection at these loci (Table 2). Homozygosity (*p* > 0.05) was detected in HLA ∼B, ∼C, ∼DRB1 and ∼DQA1 (Table 2).

**TABLE 1:** HWE parameters for high resolution typing. Exact Test using Markov chain for all loci with 100 000 dememorization steps.

Locus	#Genotypes	Obs Het	Exp Het	p-HWE
HLA ∼A	111	0.07207	0.96714	<0.0001**
HLA ∼B	345	0.27536	0.95592	<0.0001**
HLA ∼C	128	0.03906	0.86489	<0.0001**
HLA ∼DRB1	1927	0.10223	0.94003	0.0015*
HLA ∼DQA1	104	0.12500	0.71363	<0.0001**
HLA ∼DQB1	325	0.55077	0.93905	<0.0001**

#Genotypes, number of genotypes; Obs Het, observed heterozygosity; Exp Het, expected heterozygosity; p-HWE, *p*-value for HWE deviation; ** highly significant (*p* < 0.0001); * significant (*p* < 0.01) difference between observed and expected heterozygosity.

**TABLE 2:** Slatkin’s implementation of Ewens-Watterson homozygosity test of neutrality (Slatkin, 1994;^1996^). Observed homozygosity (homozygosity F statistic ∼ a sum of squared allele frequencies) compared to expected homozygosity (simulated under neutrality/equilibrium expectations for the same sample taking into account unique alleles).

Locus	Observed F	Expected F	Variance in F	Fnd	Fp
HLA ∼A	0.0362	0.0657	0.0003	−1.7622	<0.0001**
HLA ∼B	0.0461	0.0367	0.0001	1.2062	0.8965
HLA ∼C	0.1385	0.1496	0.0026	−0.2165	0.5070
HLA ∼DRB1	0.0602	0.0446	0.0001	1.3792	0.9163
HLA ∼DQA1	0.2898	0.4738	0.0262	−1.1368	0.0960
HLA ∼DQB1	0.0626	0.1091	0.0013	−1.3042	0.0133*

Observed F (observed homozygosity F statistic); Expected F (expected homozygosity F statistic); Fnd (Normalised deviate of F statistic); Fp (*p*-value F statistic); ** highly statistically significant (*p* < 0.0001); * significant (*p* < 0.05).

### Allele Frequencies

**FIGURE 1:**
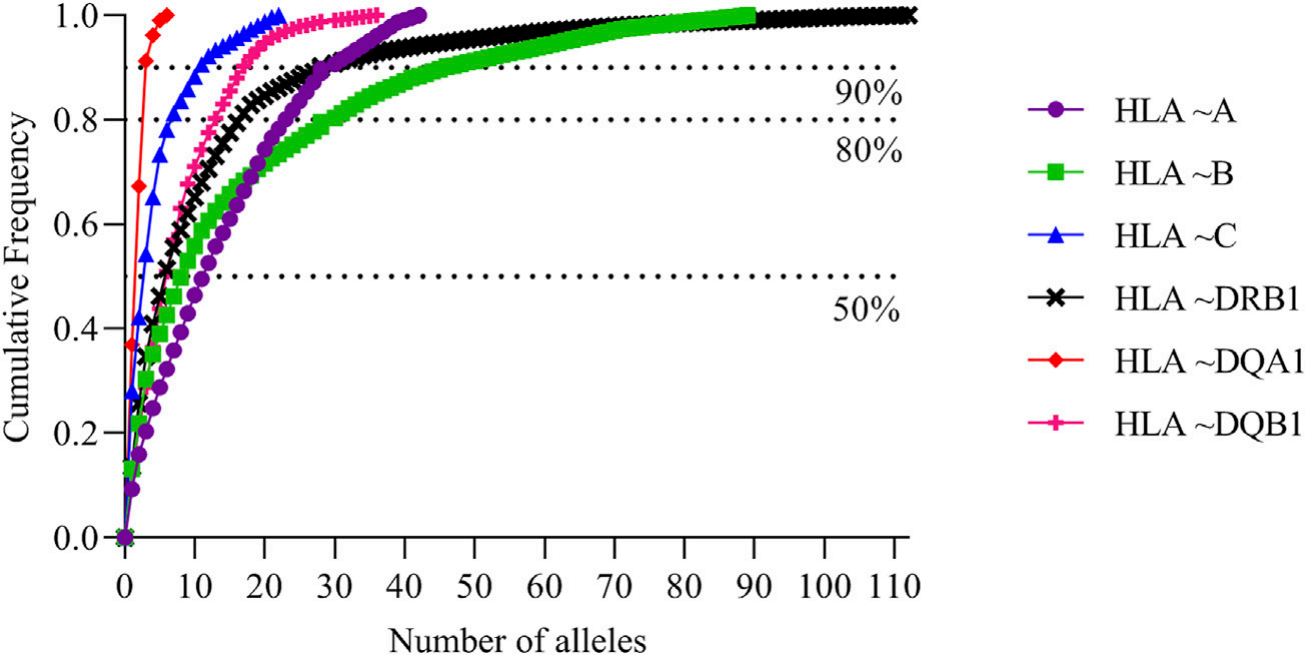
South African cumulative allele frequencies. Cumulative allele frequencies indicating population coverage of South African HLA ∼A, ∼B, ∼C, ∼DRB1 and ∼DQB1 alleles. HLA alleles were sorted according to their allele frequencies in descending order; cumulative frequencies were plotted according to the number of alleles.

The full list of alleles is detailed in Supplementary Table S1 which includes all typing frequencies from the South African population. The top 20 most frequent alleles across the different loci are summarized in Table 3. HLA ∼DQA1*05:02 (0.258), ∼DQA1*04:02 (0.194) and ∼ C*17:01 (0.149) were the three most common alleles in our dataset (Table 3). From the 3005 individuals in our data set, complete HLA data for each locus were as follows: HLA ∼A (111), HLA ∼B (345), HLA ∼C (128), HLA ∼DRB1 (1927), HLA ∼DQA1 (104) and HLA ∼DQB1 (325). There was profound data missingness which we attempted to address in our quest to highlight HLA diversity from South African populations. Figure 1 summarizes the cumulative allele frequencies from the South African populations described in this study. We additionally include PhyloD generated (Listgarten et al., 2008) HLA ∼A, ∼B and ∼C estimated genotypes (with probabilities) and allele frequencies in Supplementary Table S2 for population comparison and as a future resource for other researchers.

**TABLE 3:** Top 20 HLA alleles by locus and typing resolution (Full list in Supplementary Table S1).

Four-digit	
Locus	Frequency
DQA1*05:02	0.258
DQA1*04:02	0.194
C*17:01	0.149
DQA1*02:01	0.145
A*43:01	0.107
C*16:01	0.087
DQB1*03:19	0.087
DRB1*15:03	0.081
DRB1*15:01	0.071
DQB1*03:01	0.068
B*15:10	0.067
DQB1*05:03	0.063
C*16:02	0.063
C*03:04	0.062
DQB1*06:03	0.061
B*15:01	0.060
DQB1*05:02	0.058
C*14:02	0.051
DQB1*06:02	0.049
DQB1*02:01	0.047

### Haplotype Frequencies and Linkage Disequilibrium

The most common estimated two, three and four loci haplotypes were A*02:05∼C*14:02 (0.500), A*30:02∼B*45:01∼DRB1*15:03 (1.00) and A*30:02∼B*45:01∼DRB1*15:03∼DQB1*05:01 (0.500), respectively, as summarized in Table 4 and Supplementary Table S3. PyPop version 0.7.0 (Lancaster et al., 2007) could not estimate any five and six loci haplotypes at high resolution (Supplementary Table S3) due to lack of data after filtering. Pairwise LD measured by Hedrick’s D′ (Hedrick, 1987) and Cramer’s V Statistic (W_n_) (Cramér, 1946) were strongly significant (*p* < 0.0001) and significant (*p* < 0.05) except for C:DQB1 loci pairs (Table 5).

**TABLE 4:** The twenty most frequent two, three, four, five and six loci haplotype frequencies (Full list in Supplementary Table S3). No data was available after filtering to compute five and six loci haplotype frequencies in Pypop (Lancaster et al., 2007). Only 18 four∼loci haplotypes were identified.

Two loci	HF	Three loci	HF	Four loci	HF
A*02:05∼C*14:02	0.500	A*30:02∼B*45:01∼DRB1*15:03	1.000	A*30:02∼B*45:01∼DRB1*15:03∼DQB1*05:01	0.500
A*29:02∼C*17:01	0.500	DRB1*11:02∼DQA1*05:02∼DQB1*03:19	1.000	A*30:02∼B*45:01∼DRB1*15:03∼DQB1*06:02	0.500
C*17:01∼DQA1*04:02	0.579	C*17:01∼DRB1*11:02∼DQB1*03:19	0.667	B*42:01∼C*17:01∼DRB1*15:03∼DQA1*04:02	0.571
B*42:01∼DQA1*04:02	0.556	B*42:01∼C*17:01∼DQA1*04:02	0.657	B*42:02∼C*17:01∼DRB1*11:02∼DQB1*03:19	0.333
A*23:01∼DQA1*02:01	0.500	A*23:01∼DQA1*02:01∼DQB1*02:01	0.500	B*15:10∼C*17:01∼DRB1*11:02∼DQB1*03:19	0.167
A*80:01∼DQA1*02:01	0.500	A*80:01∼DQA1*02:01∼DQB1*02:01	0.500	B*52:02∼C*03:04∼DRB1*11:02∼DQB1*03:19	0.167
B*42:01∼C*17:01	0.406	B*42:01∼DRB1*15:03∼DQA1*04:02	0.444	B*41:02∼C*17:01∼DRB1*11:02∼DQB1*03:19	0.167
C*17:01∼DQB1*03:19	0.313	C*17:01∼DRB1*15:03∼DQA1*04:02	0.444	B*41:02∼C*17:01∼DRB1*15:03∼DQB1*03:19	0.167
C*17:01∼DQB1*04:01	0.313	A*30:02∼B*45:01∼DQB1*05:01	0.400	B*42:01∼C*17:01∼DRB1*11:02∼DQA1*04:02	0.143
A*30:02∼DRB1*15:03	0.267	A*30:02∼B*45:01∼DQB1*06:02	0.400	B*42:01∼C*17:01∼DRB1*03:02∼DQA1*04:02	0.071
DQA1*02:01∼DQB1*02:01	0.222	C*17:01∼DQA1*04:02∼DQB1*04:01	0.313	B*57:03∼C*17:01∼DRB1*03:02∼DQA1*04:02	0.071
C*03:04∼DQA1*05:02	0.218	C*17:01∼DQA1*05:02∼DQB1*04:01	0.312	B*15:10∼C*17:01∼DRB1*15:03∼DQA1*05:02	0.071
A*30:02∼B*45:01	0.211	A*30:02∼DRB1*15:03∼DQB1*05:01	0.250	B*42:02∼C*03:04∼DRB1*15:03∼DQA1*05:02	0.071
A*30:02∼DQB1*06:02	0.208	A*30:02∼DRB1*15:03∼DQB1*06:02	0.250	B*42:01∼C*17:01∼DQA1*04:02∼DQB1*04:01	0.345
DRB1*15:03∼DQA1*02:01	0.207	A*68:01∼DRB1*03:01∼DQB1*02:01	0.250	B*15:10∼C*03:04∼DQA1*05:02∼DQB1*04:01	0.220
B*42:02∼C*17:01	0.188	A*68:01∼DRB1*11:01∼DQB1*03:01	0.250	B*42:01∼C*17:01∼DQA1*05:02∼DQB1*04:01	0.155
C*03:04∼DQB1*04:01	0.188	B*42:01∼C*17:01∼DQB1*04:01	0.250	B*15:10∼C*03:04∼DQA1*04:02∼DQB1*04:01	0.155
C*17:01∼DRB1*15:03	0.178	B*42:01∼DRB1*11:02∼DQA1*04:02	0.222	B*15:10∼C*17:01∼DQA1*05:02∼DQB1*04:01	0.125
A*30:02∼DQB1*05:01	0.167	B*42:01∼C*17:01∼DRB1*15:03	0.211		
A*68:01∼DQB1*02:01	0.167	B*42:02∼DRB1*11:02∼DQB1*03:19	0.200		

haplotype frequency (HF).

**TABLE 5:** Pairwise linkage disequilibrium.

Locus pair	D'	Wn	*p*-value
A:B	0.0310	0.9501	<0.0001**
A:C	1.0000	1.000	<0.0001**
A:DRB1	1.0000	1.000	<0.0001**
A:DQA1	0.0000	0.9721	<0.0001**
A:DQB1	0.9583	0.7958	<0.0001**
A:DPB1	+	+	+
B:C	0.9842	0.8967	<0.0001**
B:DRB1	0.8110	0.7693	<0.0001**
B:DQA1	0.7458	0.6177	0.0050*
B:DQB1	0.9328	0.8895	<0.0001**
B:DPB1	+	+	+
C:DRB1	0.7771	0.6520	<0.0001**
C:DQA1	0.5335	0.5335	0.0070*
C:DQB1	0.4583	0.7253	0.1061^NS^
C:DPB1	+	+	+
DRB1:DQA1	0.5978	0.6758	0.0130*
DRB1:DQB1	0.8669	0.7042	<0.0001**
DRB1:DPB1	+	+	+
DQA1:DQB1	0.6288	0.6693	<0.0001**
DQA1:DPB1	+	+	+
DQB1:DPB1	+	+	+

D', Hedrick’s statistic (Hedrick, 1987); Wn, Cramer’s V statistic (Cramér, 1946) for global LD; **highly statistically significant (*p* < 0.0001); *significant (*p* < 0.05); ^NS^ not significant (*p* > 0.05); +, no high resolution HLA ∼DPB1 data; #, no data after filtering in Pypop.

### Population Comparison

**FIGURE 2:**
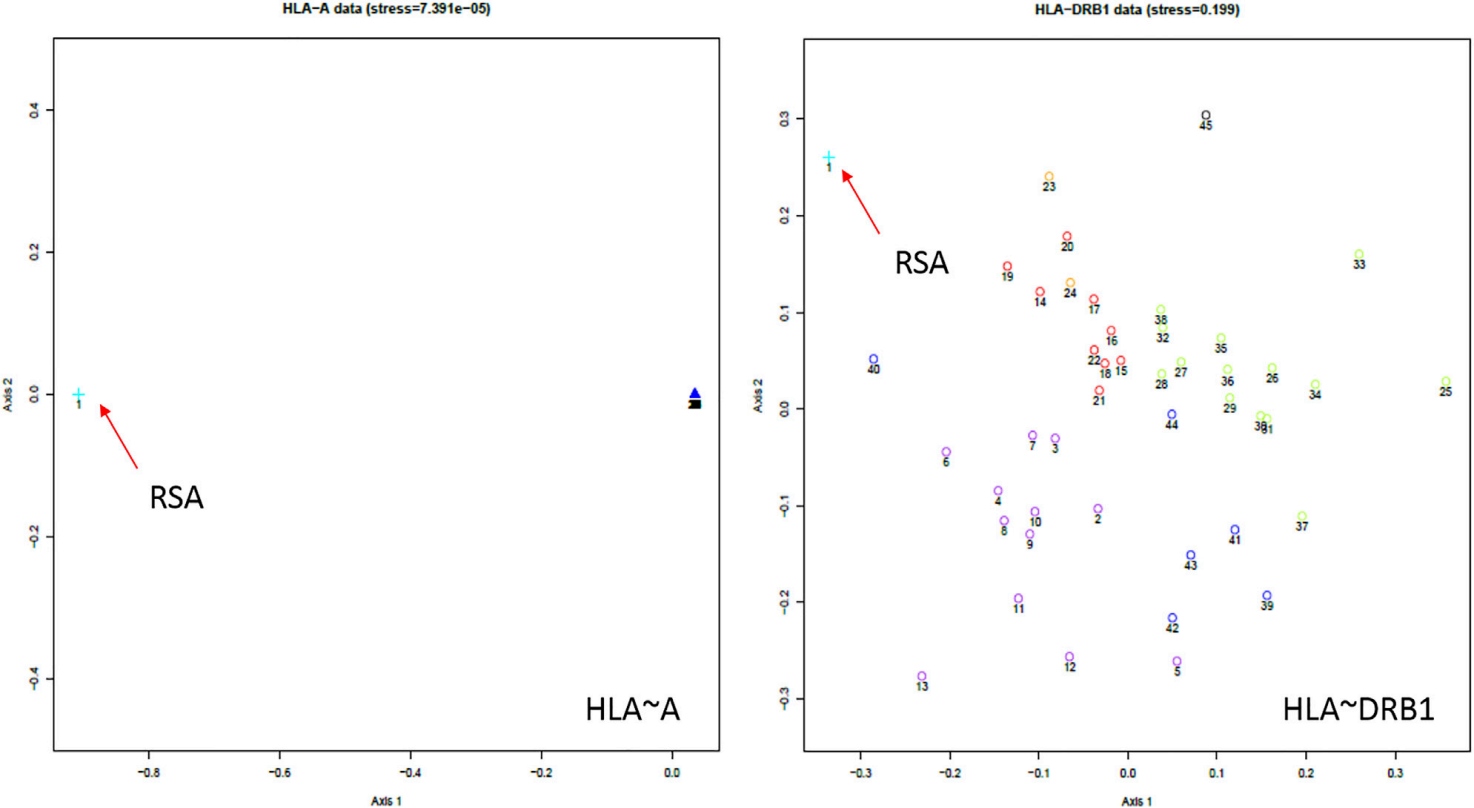
South African HLA ∼A and ∼DRB1 NMDS analysis using gene[RATE] tools (Nunes, 2016). The distances between each population correlate to the HLA profile dissimilarity in those populations. For example, in HLA ∼A, South Africans are distinctly different from the other global populations (clumped together in the far right of the HLA ∼A graph). The orientation of axes in NMDS plots is arbitrary and can be rotated in any direction. South African data = orange arrows. NMDS for all loci and description of populations compared are detailed in Supplementary Figure S1. NE-EUR (Northeast Europe), CW-EUR (Central and West Europe), SE-EUR (Southeast Europe), WASI (Western Asia), NAFR (Northern Africa), OTH (other European populations of recent origin), USER (South African). Full list in Supplementary Figure S1.

**FIGURE 3:**
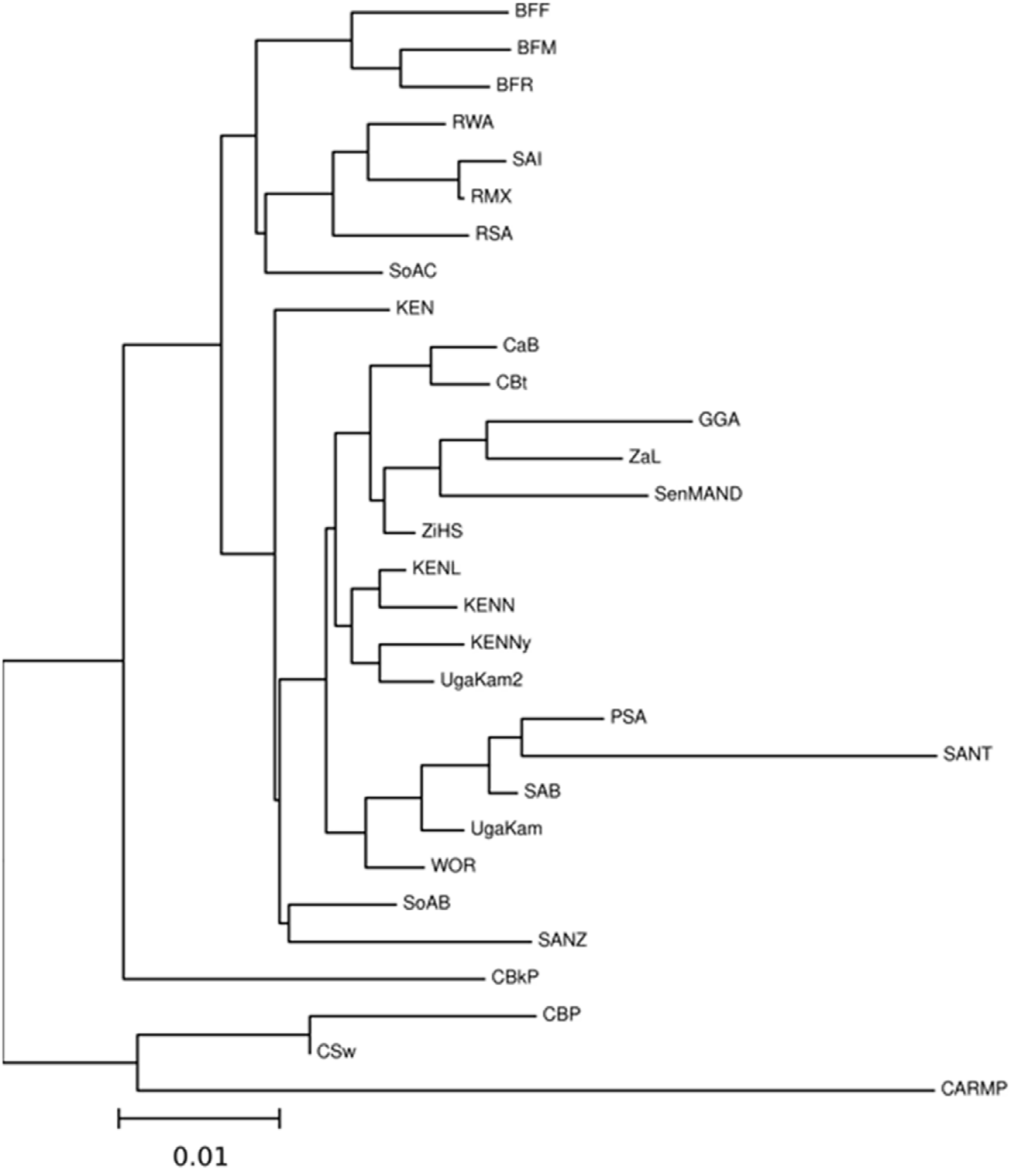
Neighbour-Joining tree based on Nei’s genetic distance for HLA ∼A, ∼B and ∼C calculated from sub-Saharan populations. High resolution (four digit typing) HLA ∼A, ∼B and ∼C allele frequencies were used to determine phylogenetic relatedness. Populations include Burkina Faso Fulani (BFF) (Modiano et al., 2001) Burkina Faso Mossi (BFM) (Modiano et al., 2001), Burkina Faso Rimaibe (BFR) (Modiano et al., 2001), Cameroon Baka Pygmy (CBP) (Torimiro et al., 2006), Cameroon Bakola Pygmy (CBkP) (Bruges Armas et al., 2003), Cameroon Bamileke (CaB) (Torimiro et al., 2006), Cameroon Beti (CBt) (Torimiro et al., 2006), Cameroon Sawa (CSw) (Torimiro et al., 2006), Central African Republic Mbenzele Pygmy (CARMP) (Bruges Armas et al., 2003), Ghana Ga-Adangbe (GGA) (Norman et al., 2013), Kenya (KEN) (Luo et al., 2002), Kenya Luo (KENL) (Cao et al., 2004), Kenya Nandi (KENN) (Cao et al., 2004), Kenya, Nyanza Province, Luo tribe (KENNy) (Arlehamn et al., 2017), PhyloD generated data (PSA) (Listgarten et al., 2008), RSA (current study), Rwanda (RWA) (Tang et al., 2000), Senegal Niokholo Mandenka (SenMAND) (Sanchez-Mazas et al., 2000), South Africa Black (SoAB) (Paximadis et al., 2012), South Africa Caucasians (SoAC) (Paximadis et al., 2012), South Africa Natal Tamil (SANT) (Hammond and Anley, 2006), South Africa Natal Zulu (SANZ) (Hammond et al., 2006), South Africa Worcester (WOR) (Grifoni et al., 2018), South African Bone Marrow Registry (SAB) (Tshabalala et al., 2018), South African Indian population (SAI) (Loubser et al., 2017a), South African Mixed ancestry (RMX) (Loubser et al., 2017b), Uganda Kampala (UgaKam) (Cao et al., 2004), Uganda Kampala pop 2 (UgaKam2) (Kijak et al., 2009), Zambia Lusaka (ZaL) (Cao et al., 2004) and Zimbabwe Harare Shona (ZiHS) (Louie et al., 2006). Current NHLS and SANBS data (RSA) showed phylogenetic relatedness to some previous South African studies i.e. SoAC (Paximadis et al., 2012), SoAB (Paximadis et al., 2012) and SANT (Hammond and Anley, 2006), but not with SANZ (Hammond et al., 2006) SAB (Tshabalala et al., 2018), SAI (Loubser et al., 2017a), RMX (Loubser et al., 2017b), and WOR (Grifoni et al., 2018) using the Nei’s genetic distances (Nei, 1972).

**FIGURE 4:**
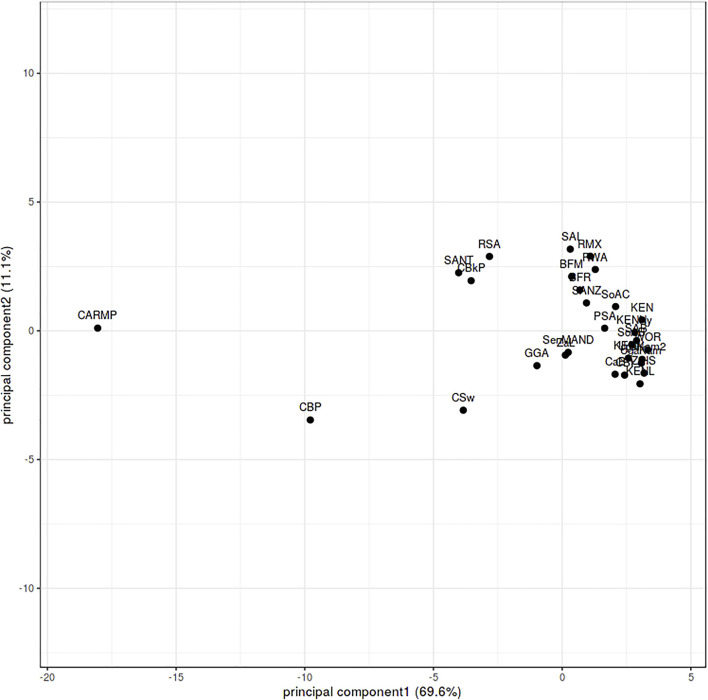
F_ST_ based principal component analysis of HLA ∼A, ∼B and ∼C calculated from sub-Saharan populations. Burkina Faso Fulani (BFF) (Modiano et al., 2001) Burkina Faso Mossi (BFM) (Modiano et al., 2001), Burkina Faso Rimaibe (BFR) (Modiano et al., 2001), Cameroon Baka Pygmy (CBP) (Torimiro et al., 2006), Cameroon Bakola Pygmy (CBkP) (Bruges Armas et al., 2003), Cameroon Bamileke (CaB) (Torimiro et al., 2006), Cameroon Beti (CBt) (Torimiro et al., 2006), Cameroon Sawa (CSw) (Torimiro et al., 2006), Central African Republic Mbenzele Pygmy (CARMP) (Bruges Armas et al., 2003), Ghana Ga-Adangbe (GGA) (Norman et al., 2013), Kenya (KEN) (Luo et al., 2002), Kenya Luo (KENL) (Cao et al., 2004), Kenya Nandi (KENN) (Cao et al., 2004), Kenya, Nyanza Province, Luo tribe (KENNy) (Arlehamn et al., 2017), PhyloD generated data (PSA) (Listgarten et al., 2008), RSA (current study), Rwanda (RWA) (Tang et al., 2000), Senegal Niokholo Mandenka (SenMAND) (Sanchez-Mazas et al., 2000), South Africa Black (SoAB) (Paximadis et al., 2012), South Africa Caucasians (SoAC) (Paximadis et al., 2012), South Africa Natal Tamil (SANT) (Hammond and Anley, 2006), South Africa Natal Zulu (SANZ) (Hammond et al., 2006), South Africa Worcester (WOR) (Grifoni et al., 2018), South African Bone Marrow Registry (SAB) (Tshabalala et al., 2018), South African Indian population (SAI) (Loubser et al., 2017a), South African Mixed ancestry (RMX) (Loubser et al., 2017b), Uganda Kampala (UgaKam) (Cao et al., 2004), Uganda Kampala pop 2 (UgaKam2) (Kijak et al., 2009), Zambia Lusaka (ZaL) (Cao et al., 2004) and Zimbabwe Harare Shona (ZiHS) (Louie et al., 2006).

**FIGURE 5:**
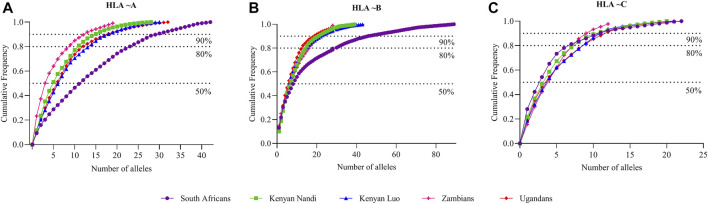
Comparison of cumulative allele frequencies from South Africa, Kenyan, Ugandan and Zambian populations. Cumulative allele frequency indicating population coverage of South African, Kenyan, Ugandan and Zambian **(A)** HLA ∼A, **(B)** ∼B and **(C)** ∼C alleles at high resolution. HLA alleles were sorted according to their allele frequencies in descending order; cumulative frequencies were plotted according to the number of alleles. HLA allele frequency data for African populations was obtained from the Allele Frequencies Net Database (AFND) (González-Galarza et al., 2015), Kenyan Luo (KENL) and Nandi (KENN) (Cao et al., 2004), Ugandan Kampala pop 2 (UgaKam2) (Kijak et al., 2009) and Zambia Lusaka (ZaL) (Cao et al., 2004).

NMDS analysis implemented in gene [RATE] tools (Nunes, 2016) suggests high genetic diversity in the HLA ∼DRB1 locus amongst the global populations referred to (Figure 2). Global populations show less diversity in HLA ∼A loci, with only two clusters (our data set and other populations) shown by NMDS (Figure 2). Additionally, our dataset distinctly clustered away from other global populations (Supplementary Figure S1). Usually, closely related populations cluster together while non-related populations form distinct clusters. Tight clusters separated from the rest suggest population sub-structure in the dataset. NMDS analysis suggests high genetic diversity in HLA ∼B, ∼DQA1, ∼DRB1, ∼DQB1 (Supplementary Figure S1). The NJ generated tree (Figure 3) shows a close relationship of the current data (RSA) with other previously described South African studies: SoAC (Paximadis et al., 2012), SoAB (Paximadis et al., 2012) and SANT (Hammond and Anley, 2006), but not with SANZ (Hammond et al., 2006), SAB (Tshabalala et al., 2018), SAI (Loubser et al., 2017a), RMX (Loubser et al., 2017b) and WOR (Grifoni et al., 2018). Interestingly, although our PhyloD generated probability simulated data (PSA) did not cluster with the data generated from RSA, it was closely related to a previous South African study, SAB (Tshabalala et al., 2018) (Figure 3). Pairwise F_ST_ based PCA showed 69.6 and 11.1% total population variability explained by PC1 and PC2, respectively (Figure 4). PCA suggests Central African Republic Mbenzele Pygmy (CARMP) are completely different from other sub-Saharan populations (Figure 4). Additional outliers include Cameroon Baka Pygmy (CBP) and Cameroon Sawa (CSw). Our data (RSA) seem to cluster together with Cameroon Bakola Pygmy (CBkP) and South Africa Natal Tamil (SANT). Our PhyloD generated PSA clustered with the other remaining populations, with Ghana Ga-Adangbe (GGA), Senegal Niokholo Mandenka (SenMAND) and Zambia Lusaka (ZaL) forming a small separate cluster (Figure 4). Cumulative frequency comparison between sub-Saharan populations in Figure 5 suggests high HLA ∼A and ∼B diversity amongst South Africans compared to others. Cumulative HLA ∼C allelic diversity in South Africans and Kenyan Nandi (KENN) (Cao et al., 2004) was more comparable to Kenyan Luo (KENL) (Cao et al., 2004), Ugandan Kampala pop 2 (UgaKam2) (Kijak et al., 2009) and Zambia Lusaka (ZaL) (Cao et al., 2004) (Figure 5).

## Discussion

This study applied several population genetic approaches to improve our understanding of HLA diversity in the South African population using retrospectively typed high resolution HLA data.

Ewens-Watterson neutrality test (Watterson, 1978) detected homozygosity (*p* < 0.0001) in HLA ∼A and ∼DRB1, which is suggestive of balancing selection at these loci (Table 2). Balancing selection is well documented to maintain HLA diversity within populations (Barreiro et al., 2008). Although the Ewens-Watterson neutrality test (Watterson, 1978) was designed for non-recombining data, the test has been evaluated to be insensitive to recombination (Zeng et al., 2007). As a result, this test may confidently be used to detect selection in HLA genes, which are known to have a high recombination rate. Deviations from neutrality due to recombination are expected to decrease haplotype homozygosity (Wright et al., 2006; Sanchez-Mazas et al., 2012a) but not influence balancing selection driven allele diversity. The exact mechanism of how balancing selection promotes HLA diversity is poorly understood (Barreiro et al., 2008). Generally, excessive homozygosity is not the result of population sub-structure, but is more common in datasets from admixed (genetically diverse) populations (Sinnock, 1975). This phenomenon has been termed the Wahlund effect (Sinnock, 1975).

The three most frequent alleles detected in South Africans have previously been reported in different AFND populations at varying frequencies (González-Galarza et al., 2015). Interestingly, HLA ∼DQA1*05:02 with a frequency of 0.258 in the current study was previously reported at lower frequencies of 0.013 and 0.004 in South African Worcester∼WOR (Grifoni et al., 2018) and Harare Zimbabwean Shona∼ZiHS (González-Galarza et al., 2015) populations, respectively. This allele has likewise been reported in African Americans at a low frequency of 0.017, and is present at an even lower frequency (0.005) in people in the Wielkopolska Region in Poland (González-Galarza et al., 2015). Additionally, our most common class I allele, HLA ∼C*17:01 (Supplementary Table S1) with a frequency of 0.149, has previously been reported at lower frequencies in other South African populations. These include South African Worcester∼WOR (Grifoni et al., 2018), black South Africans∼SoAB (Paximadis et al., 2012), Caucasian South Africans∼SoAC (Paximadis et al., 2012) and in the South African Bone Marrow Registry∼SAB (Tshabalala et al., 2018) with frequencies of 0.053, 0.111, 0.005 and 0.028, respectively. This allele (HLA ∼C*17:01) is present at lower frequencies (<0.01) in Caucasian, Asian and Hispanic populations residing in the USA, while observed at higher frequencies (>0.06) in Africans, African Americans and Caribbeans (González-Galarza et al., 2015). HLA ∼DQA1*04:02 (frequency of 0.194 and second most common in this study), has not previously been reported in any other South African study, but lower frequencies of 0.006 and 0.001 have been reported in Czech Republic (Europe) and San Diego (USA) populations, respectively (González-Galarza et al., 2015; Zajacova et al., 2016; Moore et al., 2018).

The top three haplotypes detected in the South African population have not been reported in any population in the AFND (González-Galarza et al., 2015). There was a strong global LD between all locus pairs in our study except for C:DQB1∼*p* = 0.1061 (Table 5). Haplotype diversity coupled with highly significant LD might provide insight into purifying selection (Alter et al., 2017) in the HLA genomic region. Due to data missingness, allele frequencies were computed for individual loci with all double blank alleles removed. However, for haplotype frequencies, we could not filter missing data since no data was available for computations after attempted filtering. As a result, the reported haplotype frequencies in this study might be higher than their respective allele frequencies. This limitation and the retrospective nature of the study (it is not possible to access some data that might correct this limitation) does not reduce its potential usefulness particularly given the important need for HLA data from these populations.

Population comparisons based on allele frequencies using NMDS showed distinct differences between South Africans and other, mostly European populations. This further supports high genetic diversity in Africans in general (Chen et al., 1995; Zietkiewicz et al., 1997; Jorde et al., 2000; Prugnolle et al., 2005; Disotell, 2012), with higher diversity in some HLA loci (HLA ∼B, ∼DQA1, ∼DRB1, ∼DQB1) than others. High genetic diversity was further confirmed through cumulative frequencies (Figure 1) with an increased number of alleles required to cover the same combined cumulative frequency. Cumulative frequencies for HLA ∼A, ∼B and ∼C alleles were compared with other sub-Saharan African populations including diverse Kenyans (KENN and KENL) and Ugandans (UgaKam2) (Figure 5). South Africans displayed high diversity at HLA ∼A and HLA ∼B when compared to KENN, KENL, UgaKam2 and ZaL populations while these comparator populations showed similarities in frequency between themselves. Less diverse distribution of HLA ∼C alleles is observed in South Africans and other sub-Saharan African populations. Data from the current study (RSA) was related to other South African data sets using the Nei’s’ genetic distance (Nei, 1972) and NJ method (Saitou and Nei, 1987) unrooted tree (Figure 3). We expected all the studied South African populations to cluster together, or show more phylogenetic closeness; however, this was not the case. Other South African studies including South Africa Natal Zulu ∼SANZ (Hammond et al., 2006), South African Bone Marrow Registry ∼SAB (Tshabalala et al., 2018), South African Indian ∼SAI (Loubser et al., 2017a), South African Mixed ancestry ∼RMX (Loubser et al., 2017b) and South Africa Worcester ∼WOR (Grifoni et al., 2018) were more related to other sub-Saharan populations than our current study (RSA). This is once again suggestive of high HLA diversity in South African populations, and their genetic relatedness to other African populations. Generally, if HLA data do not show the expected relatedness amongst populations (geographically, ethnolinguistically, anthropologically and linguistically related), this suggests diversification of the studied loci amongst those populations (Mack et al., 2012). Genetic distance computation assumes that genetic drift drives population differentiation, but there is strong evidence of balancing selection driving differentiation in HLA loci (Hedrick and Thomson, 1983; Hughes and Nei, 1988; Lawlor et al., 1988; Meyer and Thomson, 2001). Caution should thus be exercised when interpreting HLA genetic distance analysis between populations.

Although the expected genetic relatedness was not observed between the current study and other South African studies as mentioned above, PCA confirmed the genetic relatedness of South Africans (current RSA study) to other sub-Saharan populations (Figure 4). There is however limited high resolution data for nations neighboring South Africa for comparison, as previously reviewed (Tshabalala et al., 2015). Only data from Zambia Lusaka (ZaL) (Cao et al., 2004) and Zimbabwe Harare Shona (ZiHS) (Louie et al., 2006) was included; as a result, interpretation of this result needs to be done with caution.

The dataset had some missing alleles for some participants (data missingness). However, due to the retrospective nature of the study, we could not distinguish between missing data and blank alleles. We attempted to address this by using our dataset to simulate high resolution (four digit) class I data (Listgarten et al., 2008). Bioinformatics tools have been key in simulating high resolution typing to further understand HLA diversity (Listgarten et al., 2008; Gragert et al., 2013). There is confidence in our simulated data as it clustered with some South African HLA data (Tshabalala et al., 2018) (Figure 3) and other sub-Saharan populations (Figure 4). This provides hope in using simulated high resolution data from populations like South Africa, which currently have limited HLA data. The PhyloD tool used to address data missingness does not have an “African” representative dataset as a reference, which would provide simulations that are more accurate. Instead, an “African American” dataset was used as a reference in our simulations. We acknowledge that this reference dataset might not be ideal for all African populations since it is based on African Americans which have a particular geographic origin (west Africa). However, this is the closest available population to the South African population. Ethnic information on our study participants would have further facilitated simulating missing HLA data, but this was not available.

Highly significant deviations from HWE were observed which might be explained by the high data missingness or the presence of family members in the dataset. Other potential causes of the significant deviation from HWE include data heterogeneity, admixture, population sub-structure, a highly endogamous population and a strong selection pressure (Wills, 1991). HWE approximation may give insights into HLA genotyping quality and sampling errors. Due to the retrospective nature of the study, we acknowledge the potential of genotyping errors or failure to detect some alleles (blank allele) which might have contributed to the homozygosity observed, and which could have contributed to the deviation from HWE (Mack et al., 2012).

Additionally, the highly significant HWE deviations (as seen in this study) have been reported to influence allele and haplotype estimations (Single et al., 2002). Global LD considers all possible allele combinations from two loci studied (Klitz et al., 1995); in our case, Hedrick’s D′ (Hedrick, 1987) weights alleles in each haplotype and Cramer’s V Statistic (W_n_) (Cramér, 1946) is a multi-allelic correlation measure between pairs of loci. Haplotype frequency is influenced by LD, sample size, completeness of HLA data and allele frequency (Lewontin, 1964), especially if gamete phase is unknown (reviewed in Mack et al. (2012)). Other reported confounders to haplotype estimation include typing ambiguity (Castelli et al., 2010) and sample size (Gourraud et al., 2015).

We also note the limitation of not having access to demographic information and disease status of the study participants, as these factors contribute to HLA diversity. Although an individual’s inherited HLA genotype does not change due to disease state, continuous exposure to pathogens in a population result in increased HLA diversity over an evolutionary time period (Prugnolle et al., 2005). Generally, HLA allele frequencies provide insight into population history and not necessarily information on selection (Blagitko et al., 1997). HLA data has been widely used to understand genetic relatedness of different populations as well as demographic events in those populations (Sanchez-Mazas and Meyer, 2014). The large sample size of the current study might shed light on some demographic events in South Africa and how these relate to other sub-Saharan populations. Population allele frequencies may be used in disease association studies and provide insight into genetic relatedness (Mack et al., 2009; Romphruk et al., 2010; Sanchez-Mazas et al., 2012b). They may additionally be used to track population evolutionary processes including migration, selection and admixture (Fernandez Vina et al., 2012).

## Conclusion

We provide insight into HLA diversity in South Africans. This constitutes part of our ongoing efforts to fully understand HLA diversity in Africans, and to build a resource for future studies. Generally, HLA genetic makeup of populations provides insight into their population history including selective pressures by pathogens (Prugnolle et al., 2005), migration, admixture, and changes in population size (Parham and Ohta, 1996; Kijak et al., 2009; Buhler and Sanchez-Mazas, 2011; Sanchez-Mazas et al., 2011). Comparison of HLA data at a population level suggests genetic differences and uniqueness of South Africans relative to other global populations. We acknowledge the limitation of the retrospective nature of the data and data missingness, the imbalance of sample sizes for each locus and haplotype pairs and methodological difficulties. Despite these limitations, this study provides a unique and large HLA dataset of South Africans, which could be a useful future resource to support anthropological studies, disease association studies, population based vaccine development and donor recruitment programs.

## Study Limitations

The study had limitations accessing the demographic data of individuals, which could have been beneficial in understanding the HLA diversity in South African populations characterized by ethnic, linguistic and racial diversity. Additionally, due to the retrospective nature of the study, we could not distinguish between homozygous typing and/or missingness of one allele. For allele frequency estimation, we filtered out the data to exclude individuals who did not have data for some particular loci, as compared to haplotype estimation where the input included the whole dataset with missing data. The imbalance of sample sizes for each locus and haplotype pairs induced methodological difficulties resulting in no tallying of allele frequencies vs. haplotype frequencies. However, we believe these limitations are far outweighed by the critical importance of understanding HLA data in these populations.

## Data Availability

The original contributions presented in the study are included in the article/Supplementary Material, further inquiries can be directed to the corresponding author.

## Abbreviations

AFND, allele frequencies net database; EM, expectation maximisation; FST, population differentiation; HLA, human leukocyte antigen; HWE, hardy-weinberg equilibrium; NHLS, national health laboratory services; NJ, neighbour-joining; NMDS, non-metrical multidimensional scaling; PCA, principal component analysis; SABMR, south african bone marrow registry; SANBS, south african national blood transfusion services.
