# Supplementary material for: High Resolution HLA ∼A, ∼B, ∼C, ∼DRB1, ∼DQA1, and ∼DQB1 Diversity in South African Populations
Source: Front Genet. 2022 Mar 4;13:711944. doi: 10.3389/fgene.2022.711944 (PMC8931603; doi:10.3389/fgene.2022.711944)
Supplement: Supplementary file 2 [file DataSheet1.docx]

Supplementary Material

**Supplementary Table S1.** High resolution (four digit) HLA ~A, ~B, ~C, ~DRB1, ~DQA1 and ~DQB1 allele frequencies in 3005 typing results.

**Supplementary Table S2.** High resolution (four digit) HLA ~A, ~B, ~C genotypes and allele frequencies from PhyloD generated data~PSA ([Listgarten et al., 2008](#_ENREF_45)). The data was simulated from our dataset which had a lot of missing data.

**Supplementary Table S3.** High resolution (four digit) estimated haplotypes and their frequencies from 3005 typing results.

**Supplementary Figure S1.** High resolution (four digit) NMDS global comparison of South African HLA ~A, ~B, ~C, ~DRB1, ~DQA1, and ~DQB1 non metric multidimensional scaling analysis using gene[RATE] tools ([Nunes, 2016](#_ENREF_61)).
