# Supplementary material for: High Resolution HLA ∼A, ∼B, ∼C, ∼DRB1, ∼DQA1, and ∼DQB1 Diversity in South African Populations
Source: Front Genet. 2022 Mar 4;13:711944. doi: 10.3389/fgene.2022.711944 (PMC8931603; doi:10.3389/fgene.2022.711944)
Supplement: Supplementary file 3 [file Image1.pdf]

HLA−A data (stress=7.391e−05)

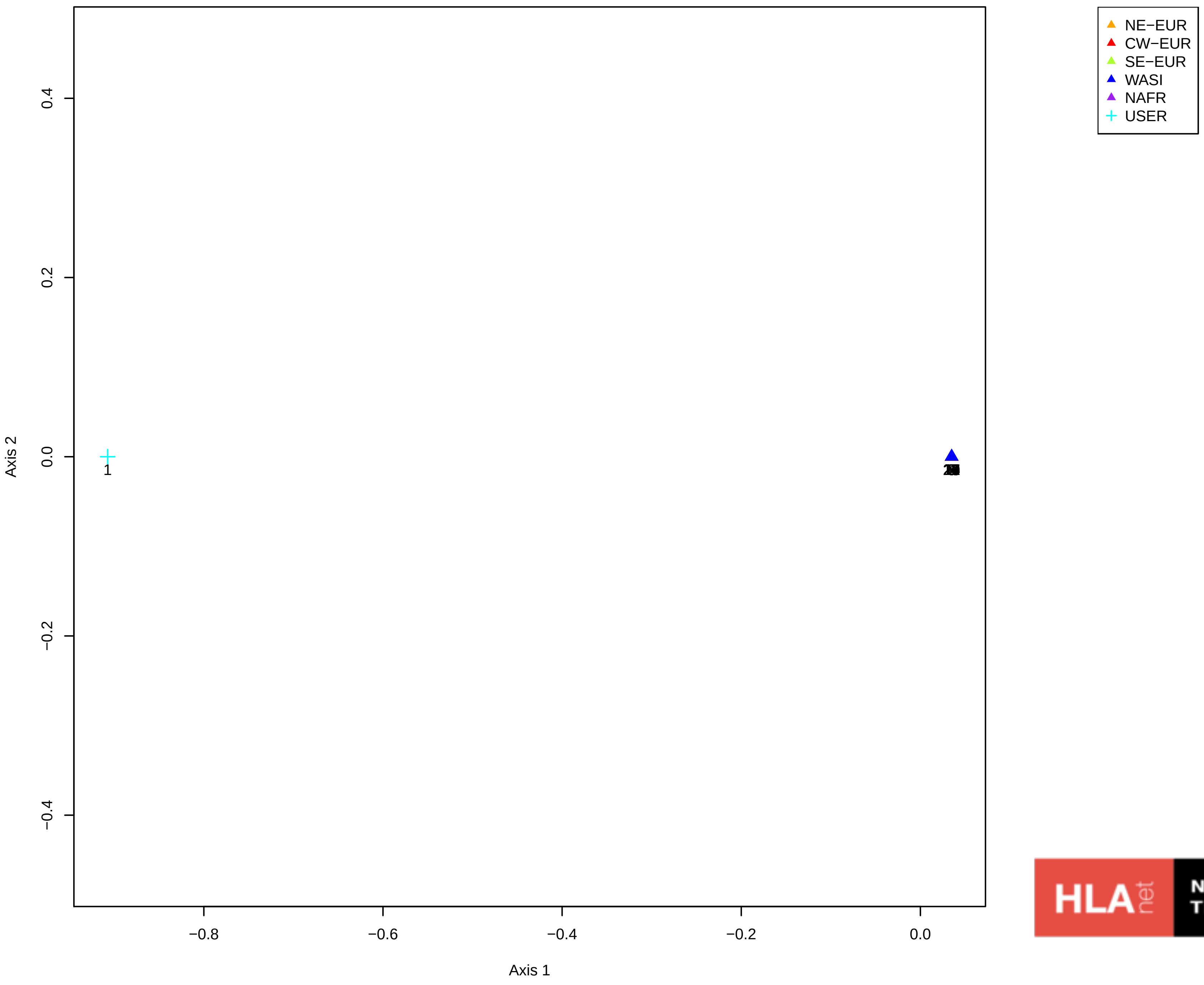

Nunes et al. (2014)
Tissue Antigens 83:307-323

1 SouthAfricans [col] (N=260)
2 Chaouya [15WS.p2] (N=74)
3 Metalsa [15WS.p13] (N=71)
4 Sudanese [16WS.p35] (N=230)
5 Austrians Oetztal [15WS.p6] (N=57)
6 British Wales [16WS.p27] (N=1000)
7 Czechs [13WS.p32] (N=105)
8 Irish [13WS.p35] (N=999)
9 Finns [13WS.p33] (N=90)
10 Russians Vologda [16WS.p15] (N=108)
11 Croatians [13WS.p31] (N=150)
12 Croatians [16WS.p37] (N=202)
13 Greek Cypriots [12WS.p166] (N=101)
14 Greeks [12WS.p34] (N=85)
15 Greeks [15WS.p12] (N=144)
16 Greeks [16WS.p44] (N=232)
17 Kosovo Albanians [16WS.p52] (N=120)
18 Druzes [13WS.p58] (N=100)
19 Georgians [13WS.p34] (N=105)
20 Indians Golla [13WS.p63] (N=88)
21 Indians New Dehli [13WS.p61] (N=66)
22 Israeli Jews [13WS.p59] (N=117)
23 Kurdish [13WS.p60] (N=30)
24 Omani [13WS.p62] (N=119)
25 Pathans [12WS.p219] (N=36)
26 Sindhi [12WS.p213] (N=39)
27 Tamil [13WS.p64] (N=50)

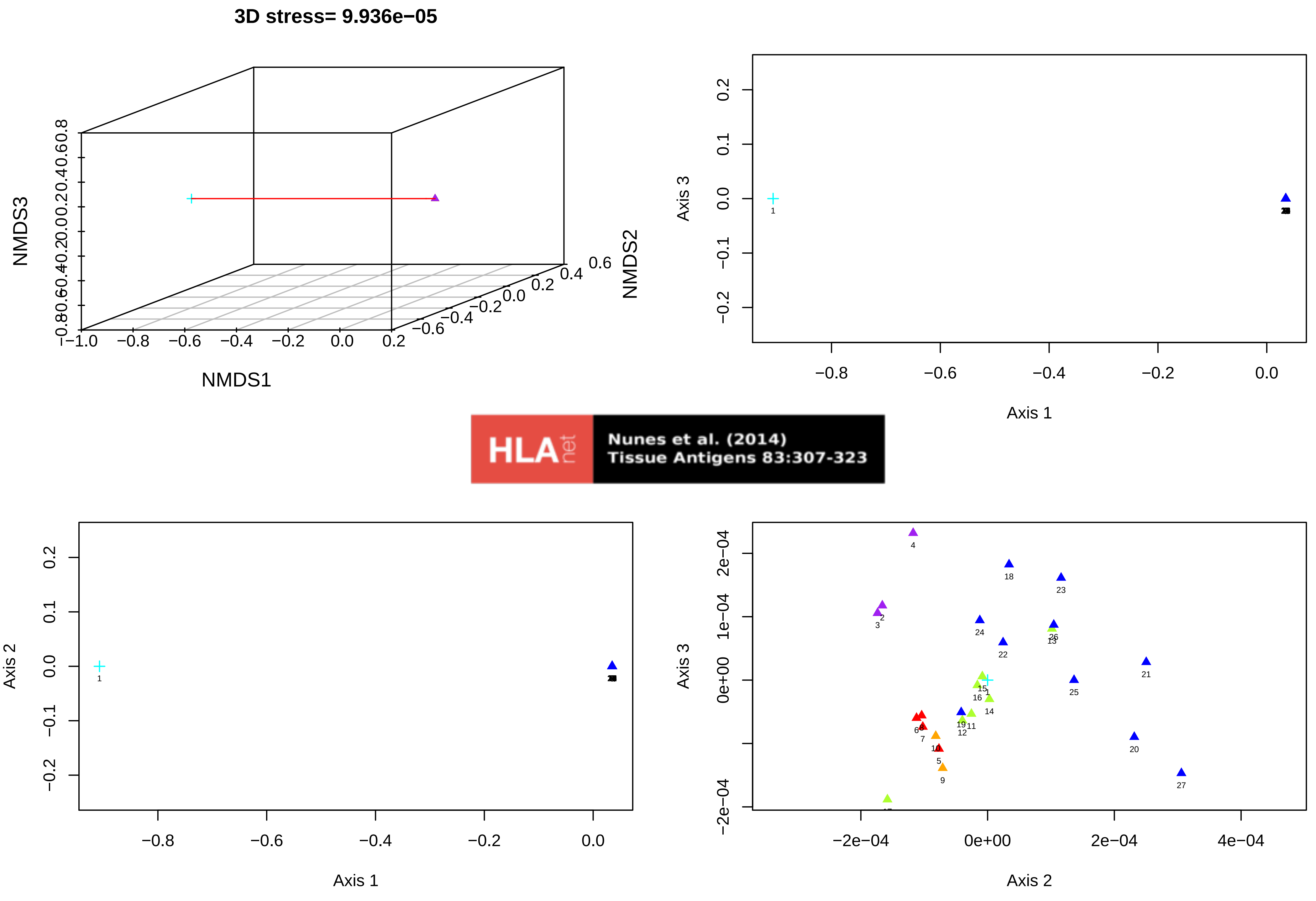

HLAnet
Nunes et al. (2014)
Tissue Antigens 83:307-323

HLA−B data (stress=0.1915)

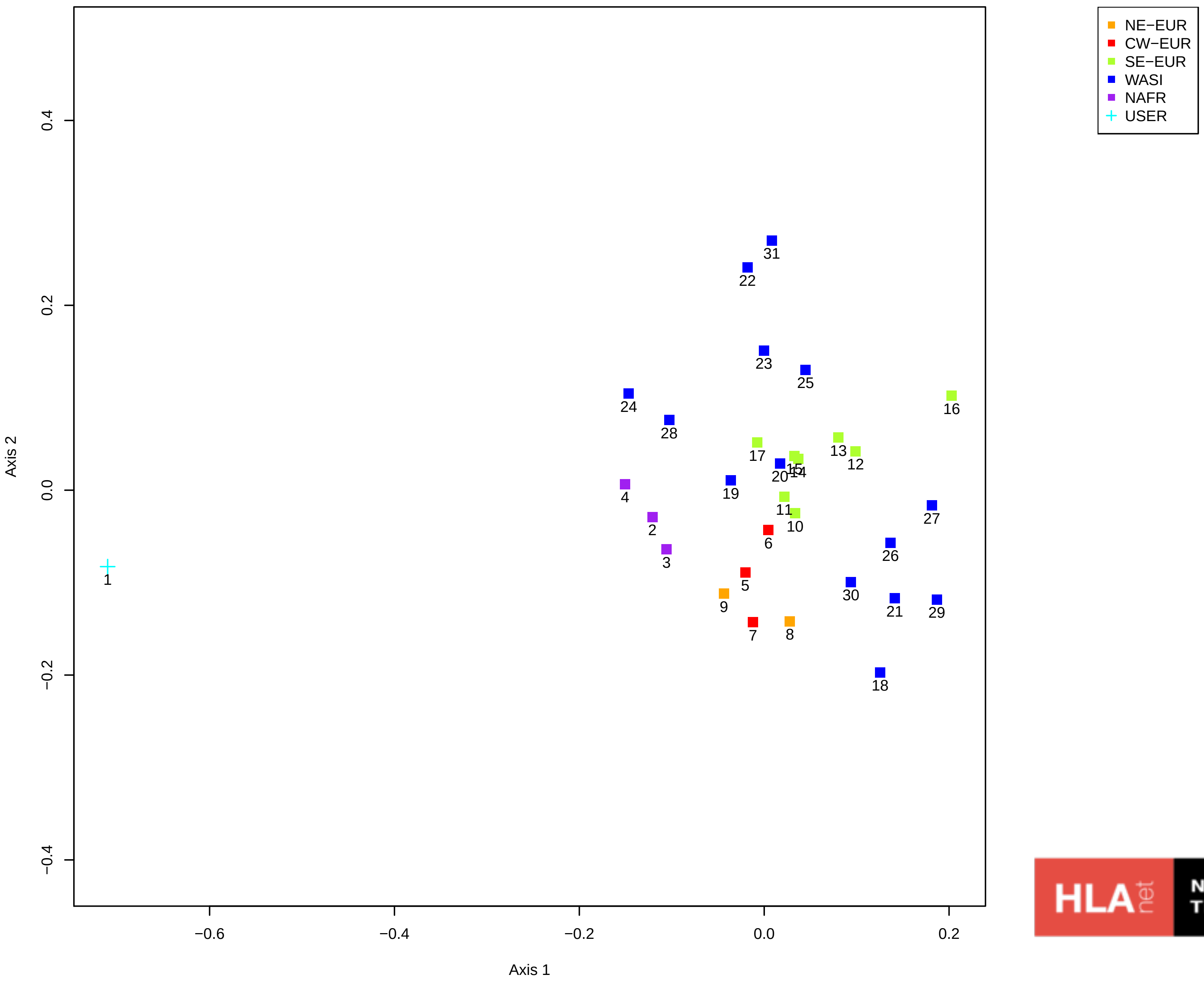

Nunes et al. (2014)
Tissue Antigens 83:307-323

1 SouthAfricans [col] (N=618)
2 Chaouya [15WS.p2] (N=73)
3 Metalsa [15WS.p13] (N=68)
4 Sudanese [16WS.p35] (N=230)
5 British Wales [16WS.p27] (N=1000)
6 Czechs [13WS.p32] (N=106)
7 Irish [13WS.p35] (N=1000)
8 Finns [13WS.p33] (N=90)
9 Russians Vologda [16WS.p15] (N=108)
10 Croatians [13WS.p31] (N=150)
11 Croatians [16WS.p37] (N=202)
12 Greek Cypriots [12WS.p166] (N=95)
13 Greeks [12WS.p34] (N=83)
14 Greeks [15WS.p12] (N=144)
15 Greeks [16WS.p44] (N=232)
16 Kosovo Albanians [16WS.p52] (N=120)
17 Macedonians [15WS.p19] (N=247)
18 Ashkenazi Jews [12WS.p116] (N=40)
19 Druzes [13WS.p58] (N=100)
20 Georgians [13WS.p34] (N=107)
21 Hunza Burushaski [12WS.p214] (N=46)
22 Indians Golla [13WS.p63] (N=104)
23 Indians New Dehli [13WS.p61] (N=66)
24 Israeli Jews [13WS.p59] (N=109)
25 Kurdish [13WS.p60] (N=29)
26 Libyan Jews [12WS.p153] (N=40)
27 Morrocan Jews [12WS.p117] (N=40)
28 Omani [13WS.p62] (N=120)
29 Pathans [12WS.p219] (N=38)
30 Sindhi [12WS.p213] (N=39)
31 Tamil [13WS.p64] (N=49)

HLA net
Nunes et al. (2014)
Tissue Antigens 83:307-323

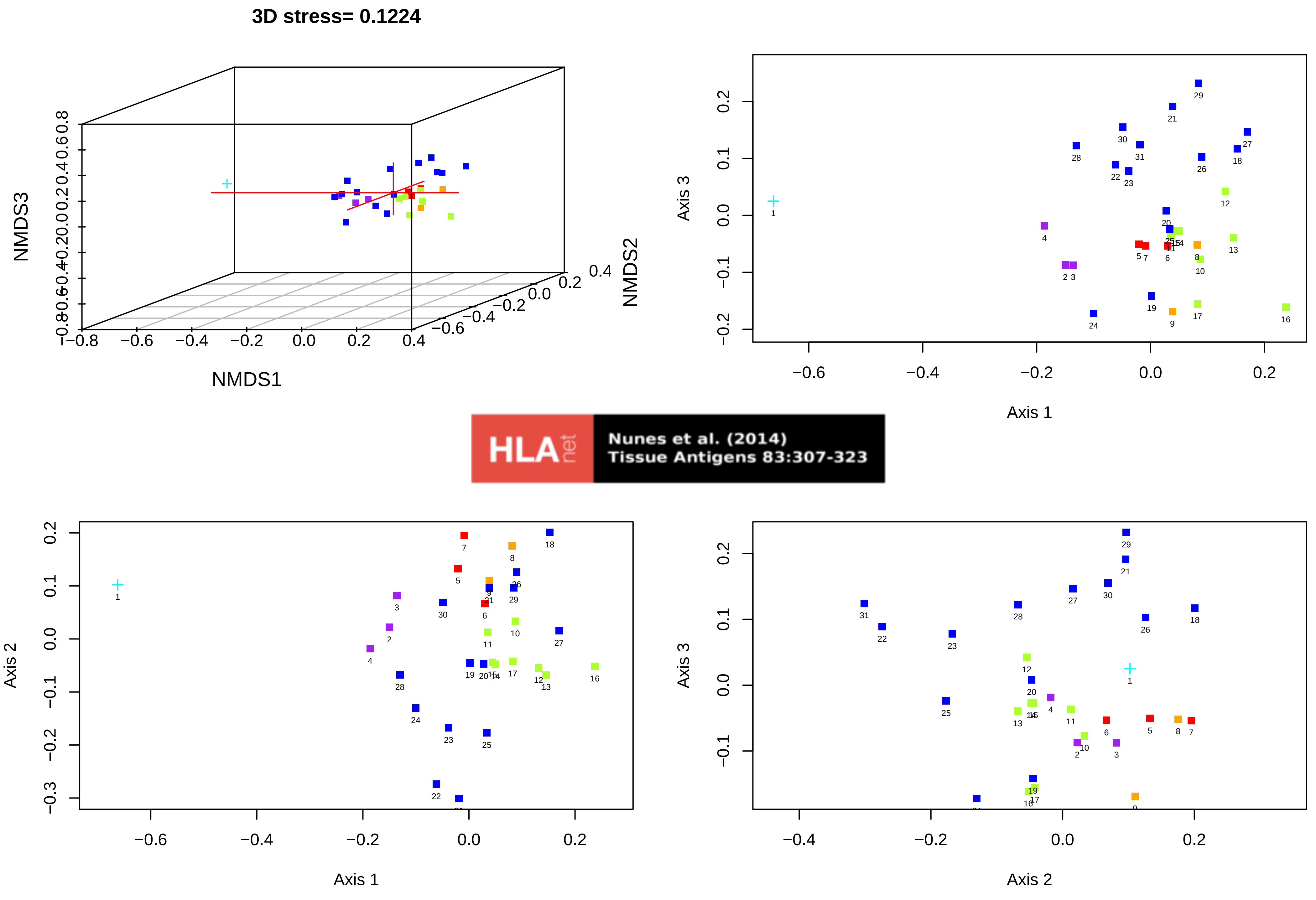

HLAnet
Nunes et al. (2014)
Tissue Antigens 83:307-323

HLA−C data (stress=8.573e−05)

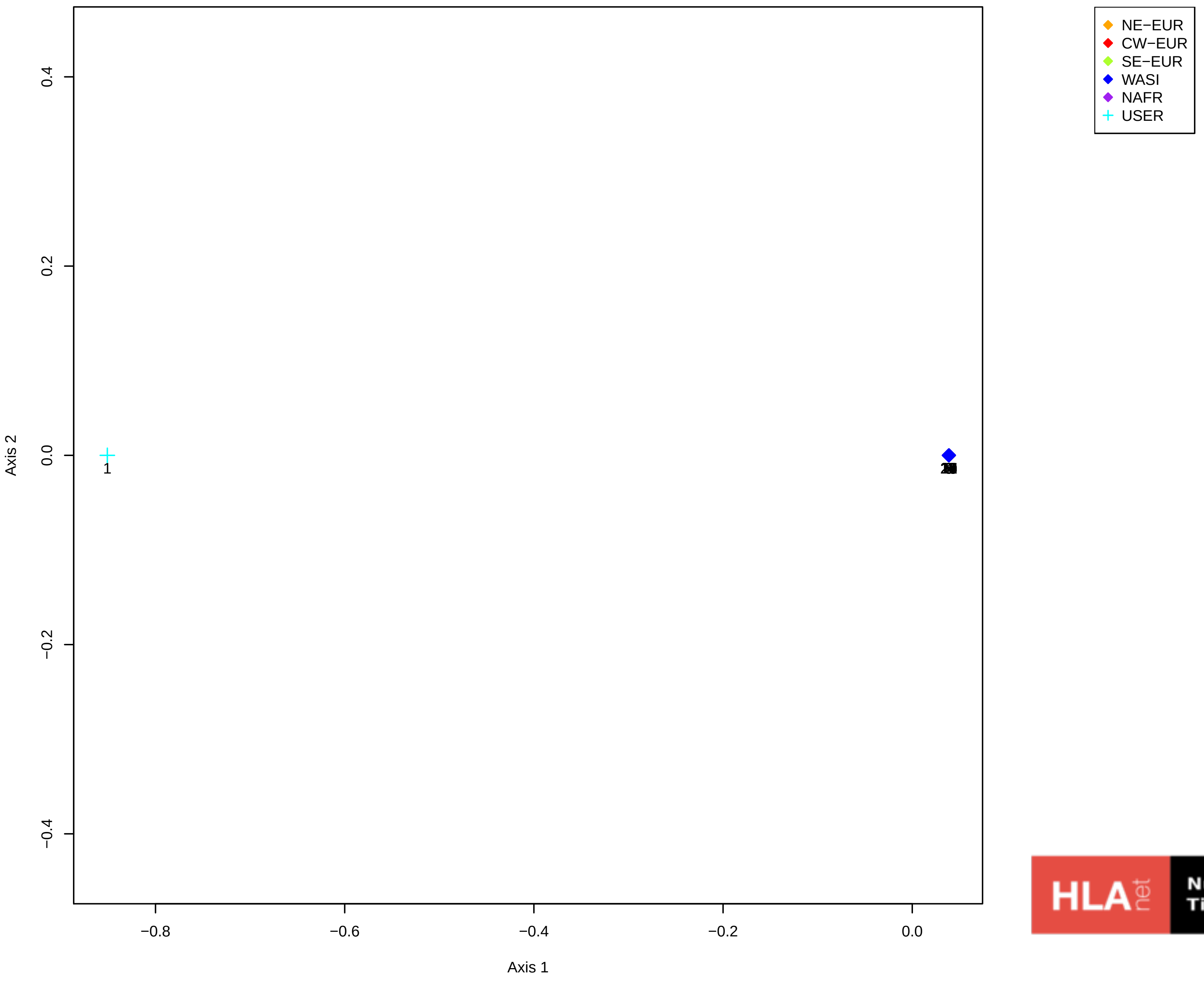

HLA net

Nunes et al. (2014)
Tissue Antigens 83:307-323

1 SouthAfricans [col] (N=144)
2 Chaouya [15WS.p2] (N=72)
3 Metalsa [15WS.p13] (N=63)
4 Sudanese [16WS.p35] (N=230)
5 British Wales [16WS.p27] (N=1000)
6 Czechs [13WS.p32] (N=105)
7 French Montpellier [16WS.p36] (N=2647)
8 Irish [13WS.p35] (N=1000)
9 Finns [13WS.p33] (N=90)
10 Albanians [15WS.p20] (N=145)
11 Greeks [15WS.p12] (N=144)
12 Greeks [16WS.p44] (N=232)
13 Kosovo Albanians [16WS.p52] (N=120)
14 Macedonians [15WS.p19] (N=250)
15 Druzes [13WS.p58] (N=100)
16 Georgians [13WS.p34] (N=107)
17 Hunza Burushaski [12WS.p214] (N=46)
18 Indians New Dehli [13WS.p61] (N=56)
19 Israeli Jews [13WS.p59] (N=94)
20 Kurdish [13WS.p60] (N=29)
21 Pathans [12WS.p219] (N=38)
22 Sindhi [12WS.p213] (N=39)
23 Tamil [13WS.p64] (N=48)

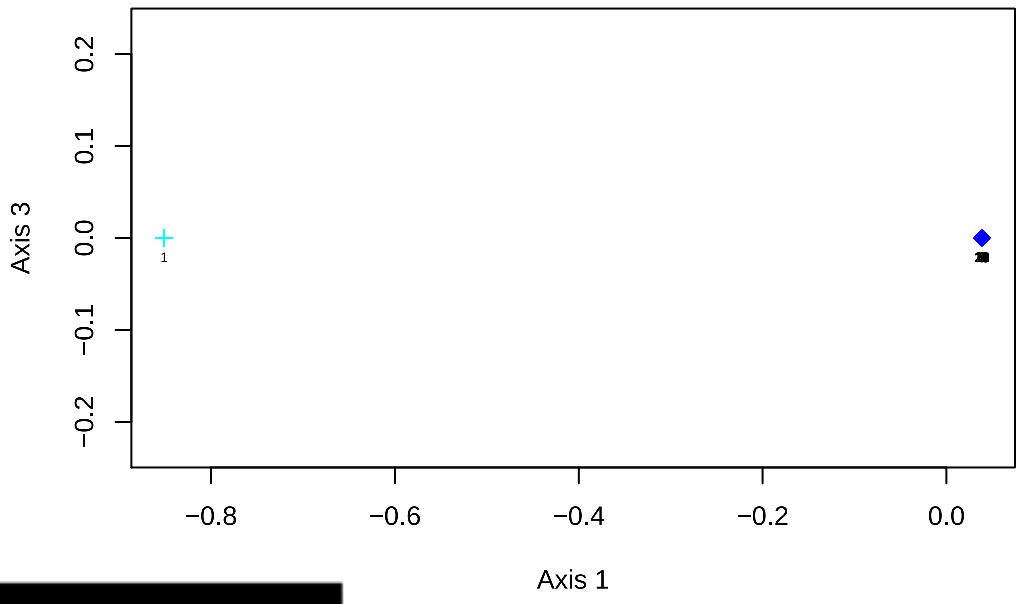

HLAnet
Nunes et al. (2014)
Tissue Antigens 83:307-323

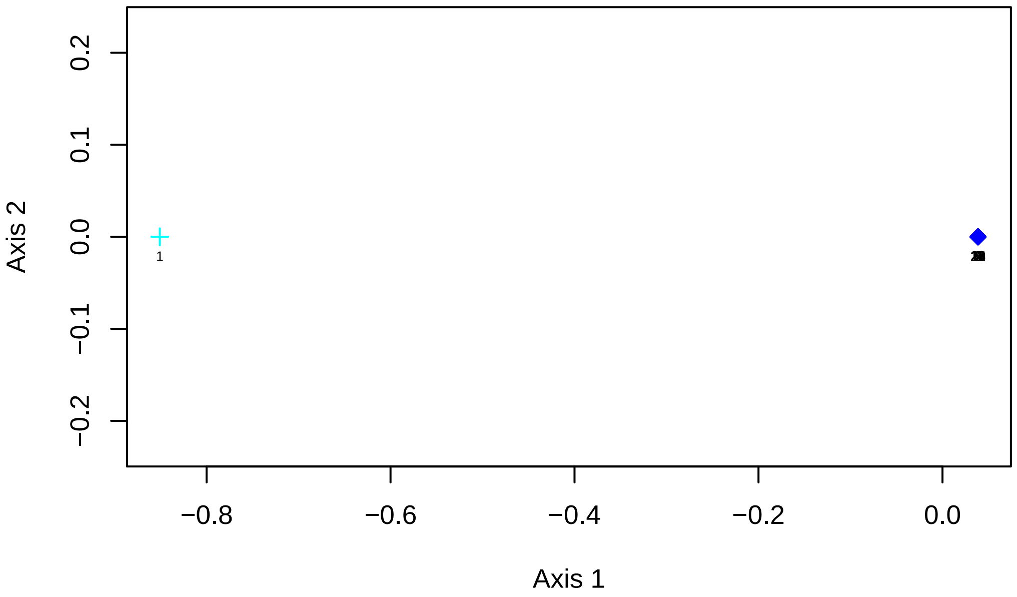

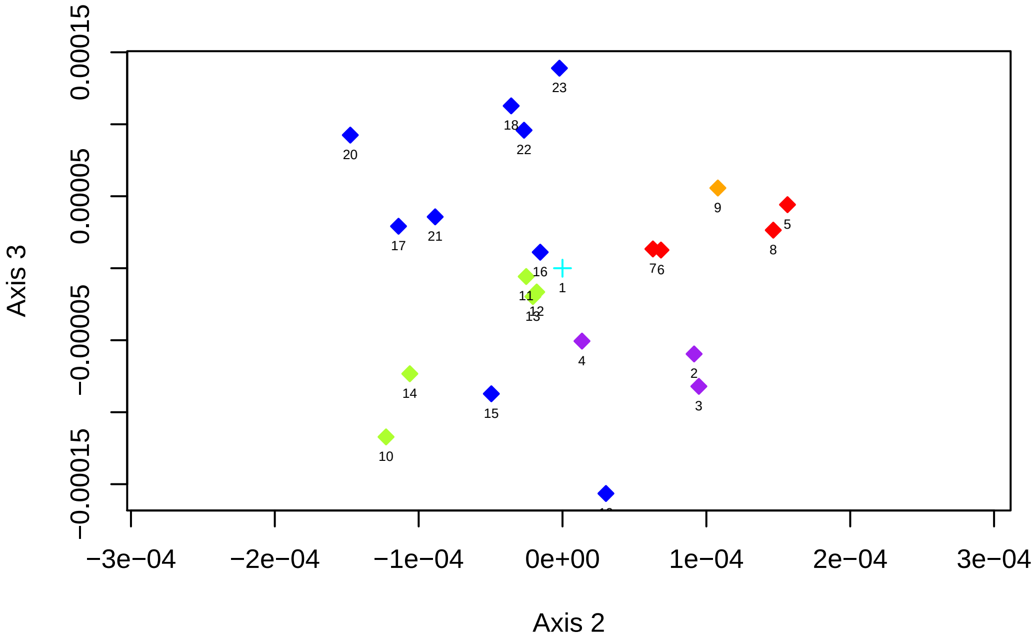

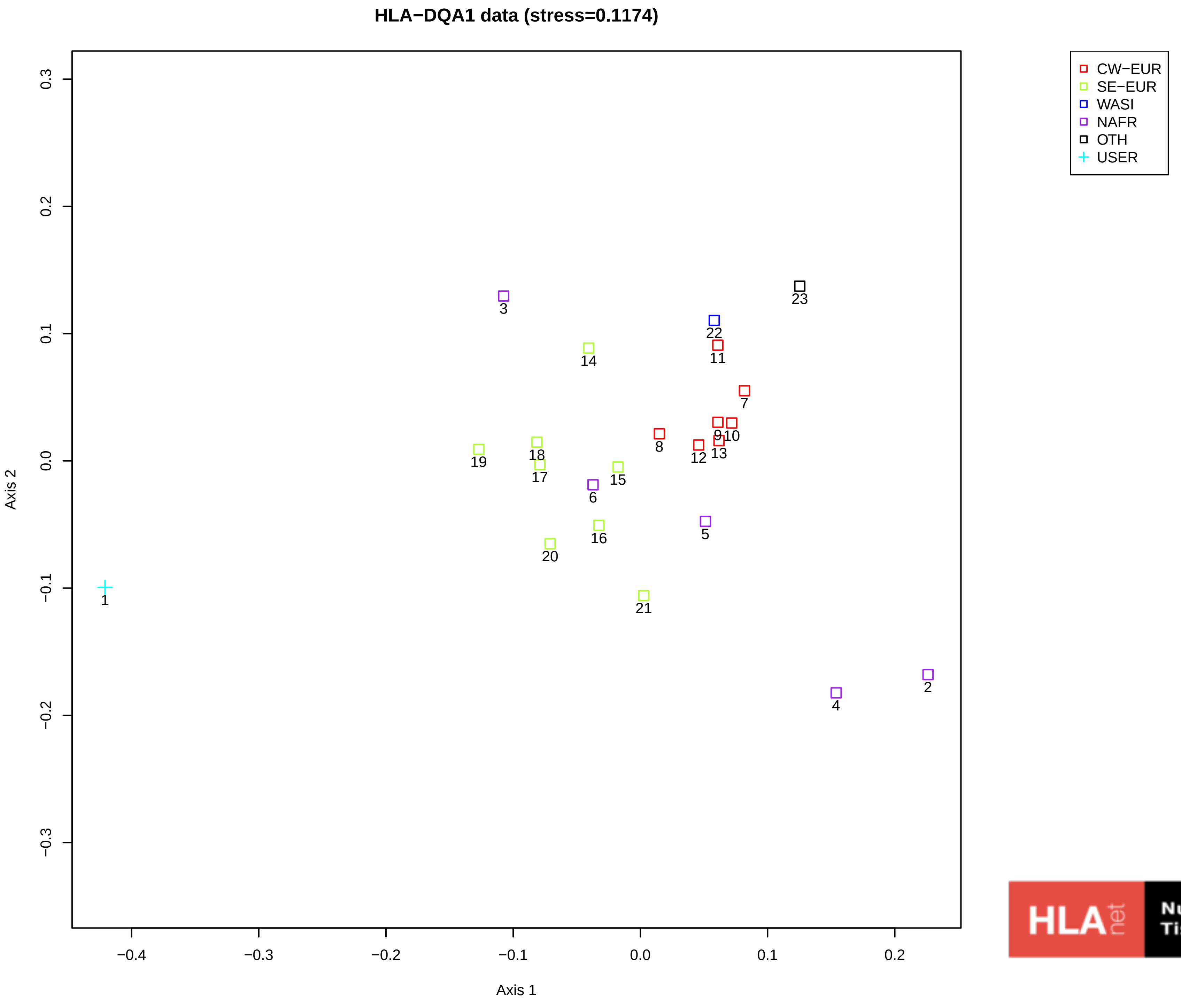

Nunes et al. (2014)
Tissue Antigens 83:307-323

1 SouthAfricans [col] (N=108)
2 Chaouya [15WS.p2] (N=83)
3 Egyptian Copts [Other.psources] (N=40)
4 Metalsa [15WS.p13] (N=89)
5 Moroccans [13WS.p20] (N=98)
6 Tunisians [Other.psources] (N=97)
7 Belgians [12WS.p158] (N=40)
8 Czechs [13WS.p32] (N=105)
9 French [12WS.p229] (N=224)
10 Polish [12WS.p156] (N=99)
11 Portuguese [12WS.p65] (N=111)
12 Spanish [12WS.p104] (N=100)
13 Spanish [12WS.p82] (N=125)
14 Croatians [12WS.p19] (N=104)
15 Croatians [12WS.p20] (N=106)
16 Greeks [12WS.p165] (N=96)
17 Italians [12WS.p42] (N=93)
18 Italians [12WS.p45] (N=99)
19 Pomaki [12WS.p164] (N=100)
20 Sardinians [12WS.p46] (N=80)
21 Slovenians [13WS.p36] (N=100)
22 Punjabi [12WS.p38] (N=51)
23 Czech Gypsies [12WS.p23] (N=33)

HLA net
Nunes et al. (2014)
Tissue Antigens 83:307-323

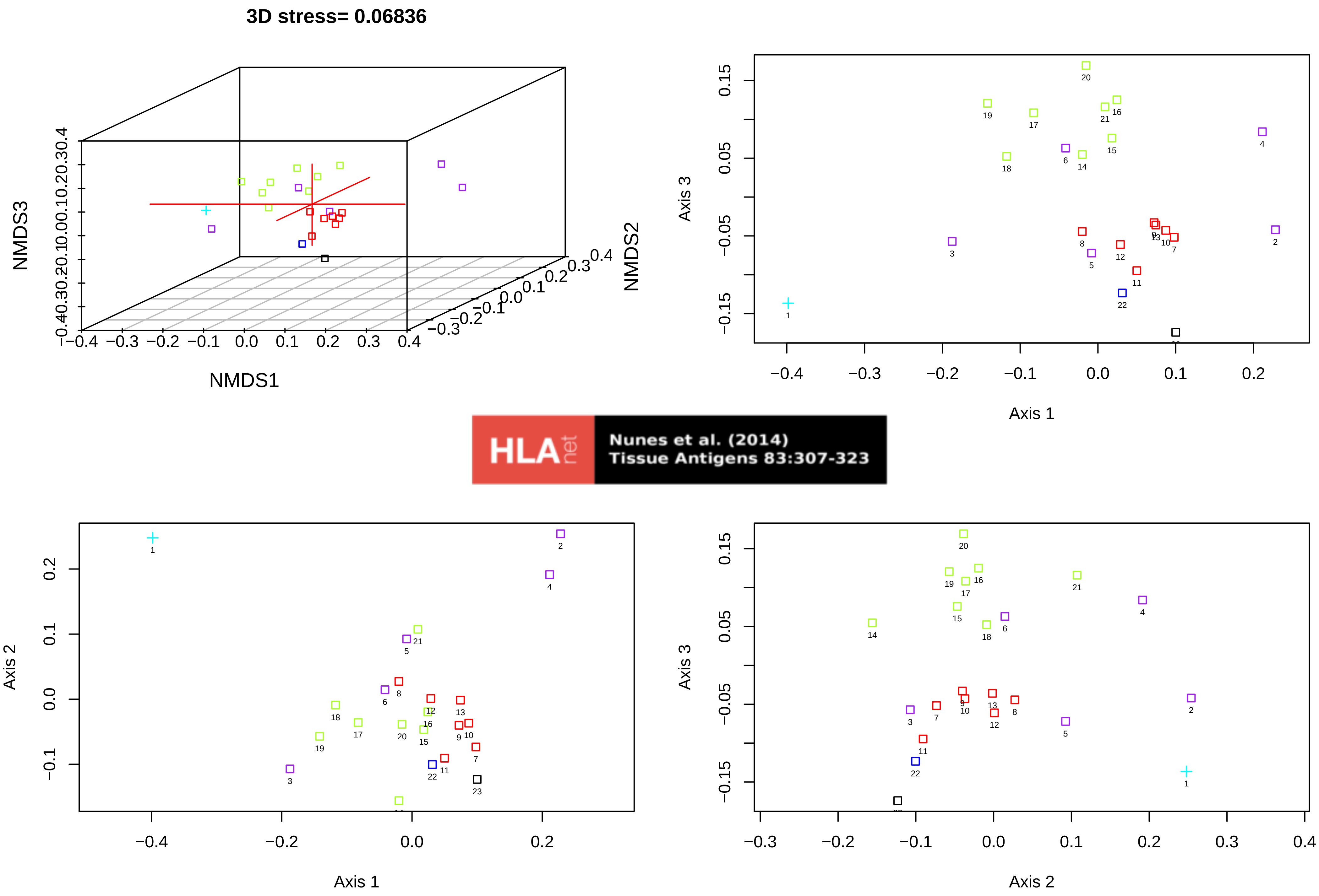
3D stress= 0.06836
NMDS1
NMDS2
NMDS3
Axis 1
Axis 3
HLAnet
Nunes et al. (2014)
Tissue Antigens 83:307-323
Axis 1
Axis 2
Axis 2
Axis 3

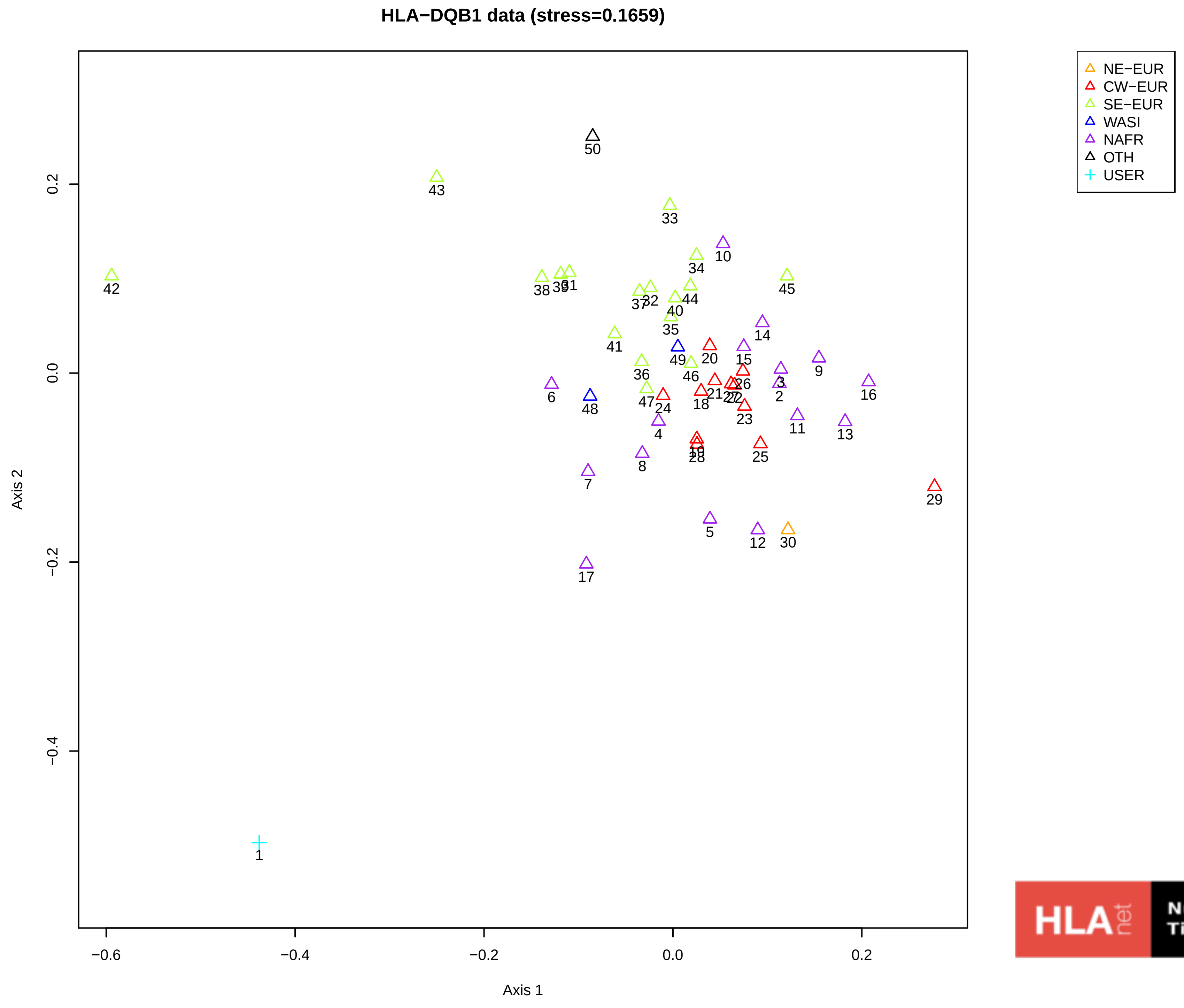

Nunes et al. (2014)
Tissue Antigens 83:307-323

1 SouthAfricans [col] (N=631)
2 Algerians [12WS.p55] (N=100)
3 Algerians [13WS.p17] (N=99)
4 Bedouins [12WS.p171] (N=98)
5 Chaouya [15WS.p2] (N=82)
6 Egyptian Copts [Other.psources] (N=40)
7 Egyptians Delta [Other.psources] (N=101)
8 Egyptians South [Other.psources] (N=94)
9 Gabes [Other.psources] (N=96)
10 Jerba [Other.psources] (N=55)
11 Matmata [Other.psources] (N=81)
12 Metalsa [15WS.p13] (N=96)
13 Moroccans [13WS.p20] (N=98)
14 Mzab [Other.psources] (N=107)
15 Sudanese [16WS.p35] (N=230)
16 Tunisians Sened [15WS.p3] (N=52)
17 Tunisians Tataouine [15WS.p4] (N=52)
18 Belgians [12WS.p158] (N=40)
19 British Wales [16WS.p27] (N=1000)
20 Czechs [12WS.p22] (N=35)
21 Czechs [13WS.p32] (N=106)
22 French [12WS.p229] (N=224)
23 French [12WS.p61] (N=234)
24 French Montpellier [16WS.p36] (N=1431)
25 Polish [12WS.p156] (N=99)
26 Portuguese [12WS.p195] (N=220)
27 Portuguese [12WS.p65] (N=111)
28 Spanish [12WS.p104] (N=100)
29 Spanish Basques [12WS.p120] (N=158)
30 Finns BMD [16WS.p39] (N=1528)
31 Albanians [15WS.p20] (N=152)
32 Bulgarians [12WS.p119] (N=120)
33 Croatians [12WS.p19] (N=102)
34 Croatians [12WS.p20] (N=104)
35 Croatians [12WS.p21] (N=139)
36 Croatians [16WS.p37] (N=202)
37 Greeks [12WS.p165] (N=96)
38 Greeks [15WS.p12] (N=144)
39 Greeks [16WS.p44] (N=232)
40 Italians [12WS.p42] (N=93)
41 Italians [12WS.p45] (N=98)
42 Kosovo Albanians [16WS.p52] (N=120)
43 Macedonians [15WS.p19] (N=218)
44 Pomaki [12WS.p164] (N=100)
45 Sardinians [12WS.p46] (N=80)
46 Slovenians [13WS.p36] (N=100)
47 Slovenians [16WS.p57 updated] (N=142)
48 Punjabi [12WS.p38] (N=42)
49 Turks [13WS.p65] (N=245)
50 Czech Gypsies [12WS.p23] (N=33)

HLA net
Nunes et al. (2014)
Tissue Antigens 83:307-323

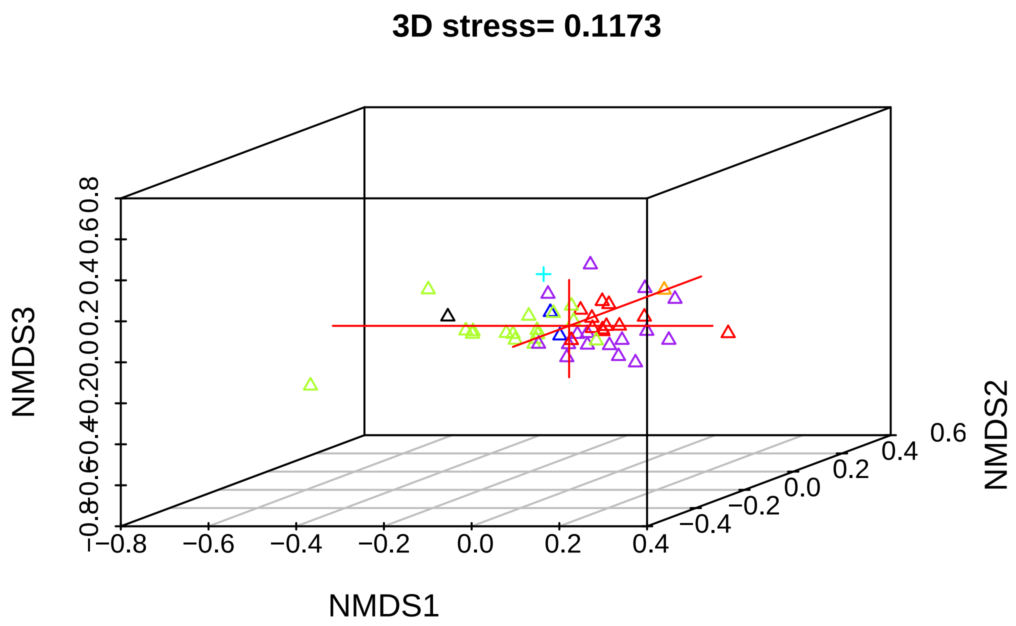

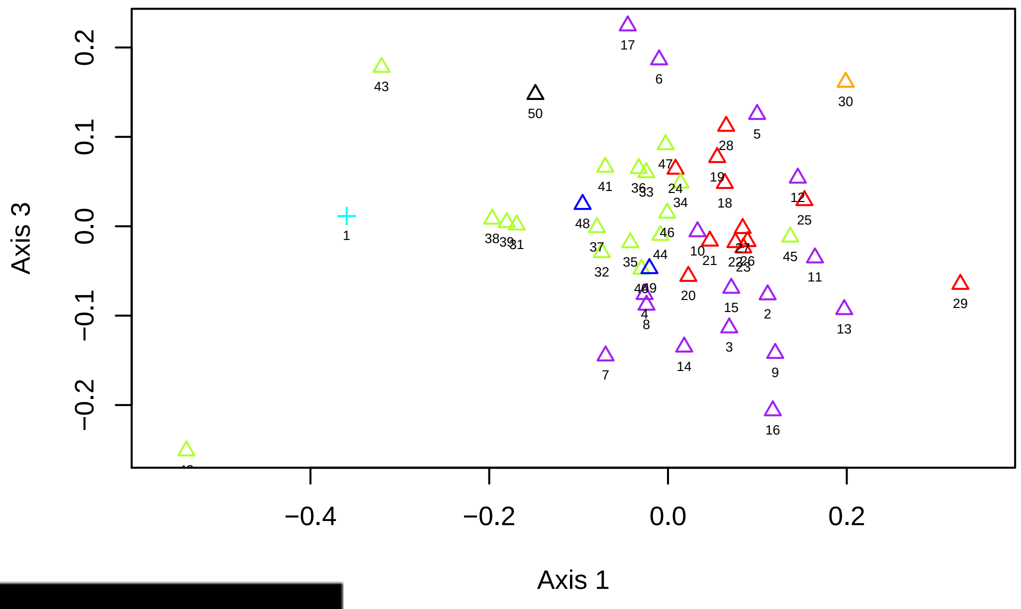

Nunes et al. (2014)
Tissue Antigens 83:307-323

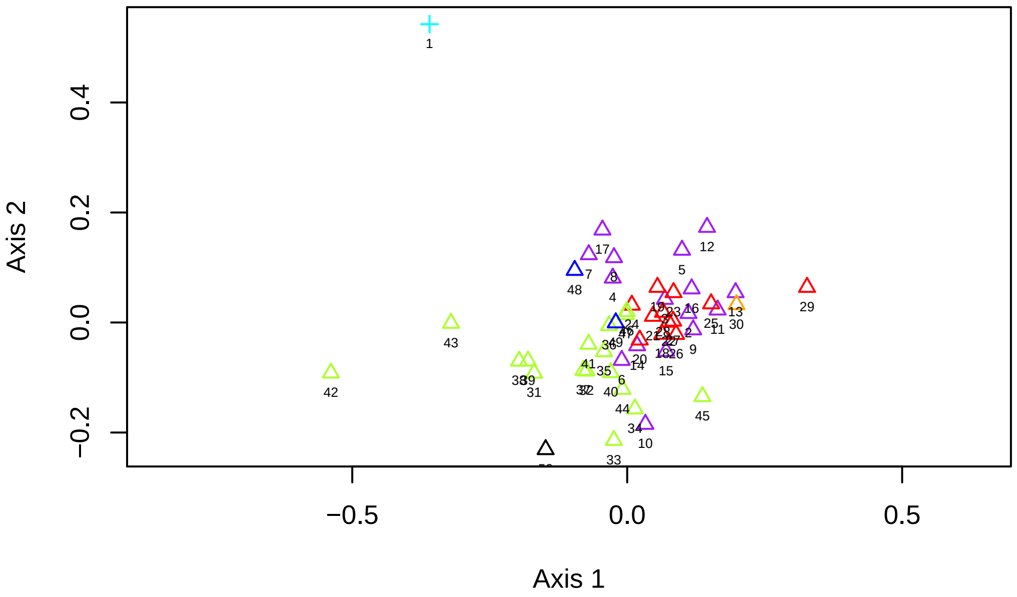

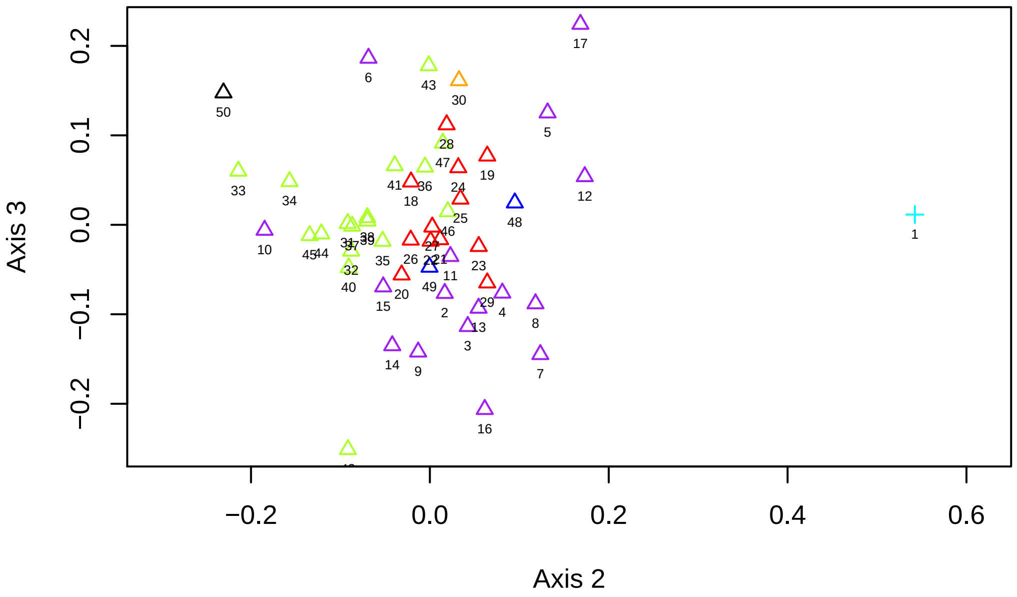

HLA−DRB1 data (stress=0.199)

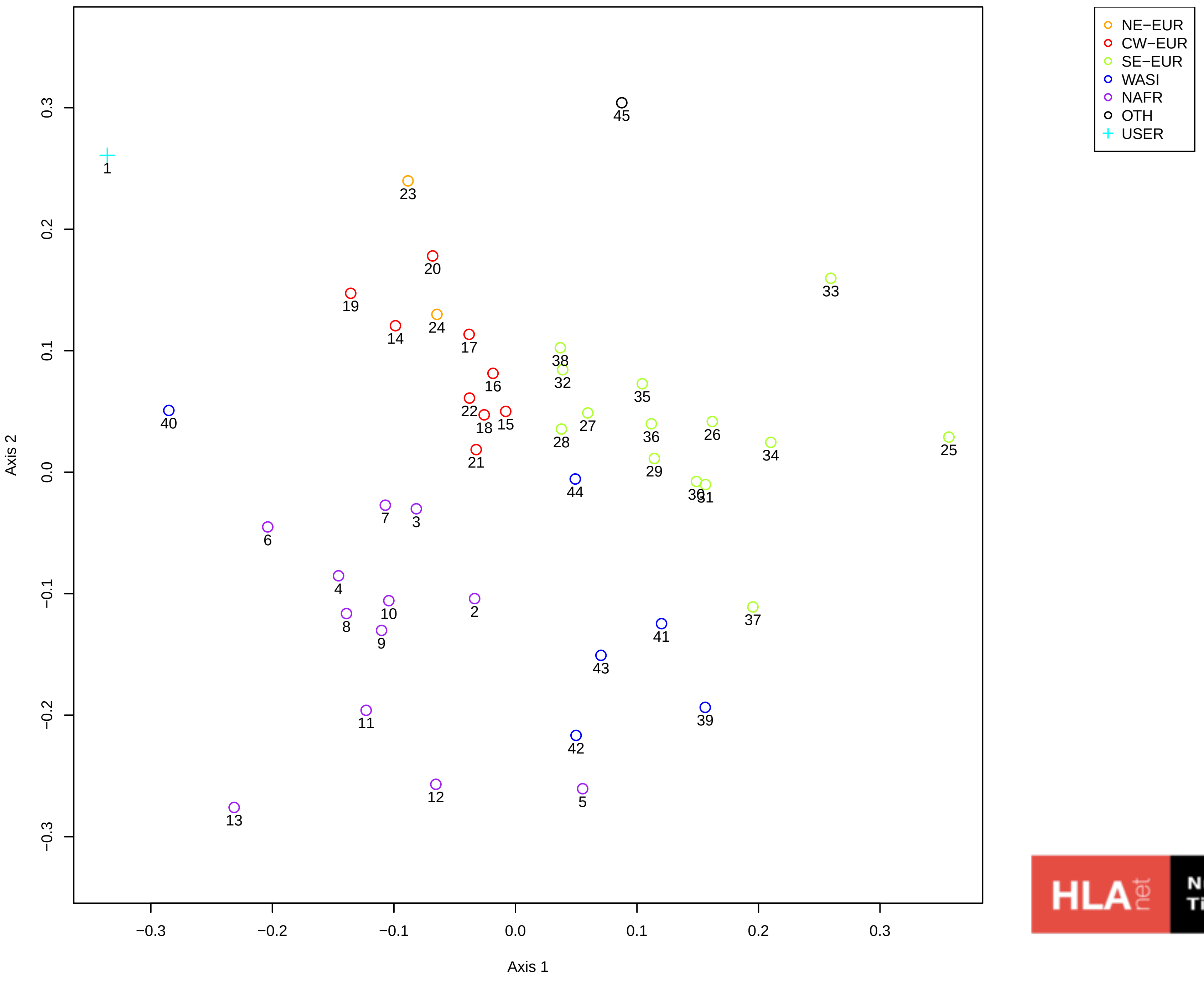

Nunes et al. (2014)
Tissue Antigens 83:307-323

1 SouthAfricans [col] (N=2274)
2 Algerians [13WS.p17] (N=99)
3 Algerians Oran [15WS.p1] (N=97)
4 Chaouya [15WS.p2] (N=99)
5 Egyptian Copts [Other.psources] (N=40)
6 Jerba [Other.psources] (N=55)
7 Matmata [Other.psources] (N=81)
8 Metalsa [15WS.p13] (N=96)
9 Moroccans [13WS.p20] (N=98)
10 Moroccans [13WS.p21] (N=91)
11 Sudanese [16WS.p35] (N=230)
12 Tunisians Sened [15WS.p3] (N=52)
13 Tunisians Tataouine [15WS.p4] (N=52)
14 British Wales [16WS.p27] (N=1000)
15 Czechs [12WS.p22] (N=36)
16 Czechs [13WS.p32] (N=103)
17 French [12WS.p61] (N=234)
18 French Montpellier [16WS.p36] (N=3902)
19 Irish [13WS.p35] (N=1000)
20 Polish [12WS.p156] (N=98)
21 Spanish [12WS.p104] (N=100)
22 Spanish [12WS.p82] (N=72)
23 Finns [16WS.p42] (N=150)
24 Russians Vologda [16WS.p15] (N=108)
25 Albanians [15WS.p20] (N=158)
26 Croatians [12WS.p19] (N=104)
27 Croatians [12WS.p21] (N=139)
28 Croatians [16WS.p37] (N=202)
29 Greeks [12WS.p165] (N=192)
30 Greeks [15WS.p12] (N=144)
31 Greeks [16WS.p44] (N=232)
32 Italians [12WS.p45] (N=99)
33 Kosovo Albanians [16WS.p52] (N=120)
34 Macedonians [15WS.p19] (N=566)
35 Pomaki [12WS.p164] (N=100)
36 Romanians [12WS.p67] (N=99)
37 Sardinians [12WS.p46] (N=80)
38 Slovenians [13WS.p36] (N=100)
39 Ashkenazi Jews [12WS.p116] (N=40)
40 Indians Golla [13WS.p63] (N=109)
41 Libanese [12WS.p145] (N=258)
42 Libyan Jews [12WS.p153] (N=40)
43 Morrocan Jews [12WS.p117] (N=40)
44 Turks [13WS.p65] (N=245)
45 Czech Gypsies [12WS.p23] (N=33)

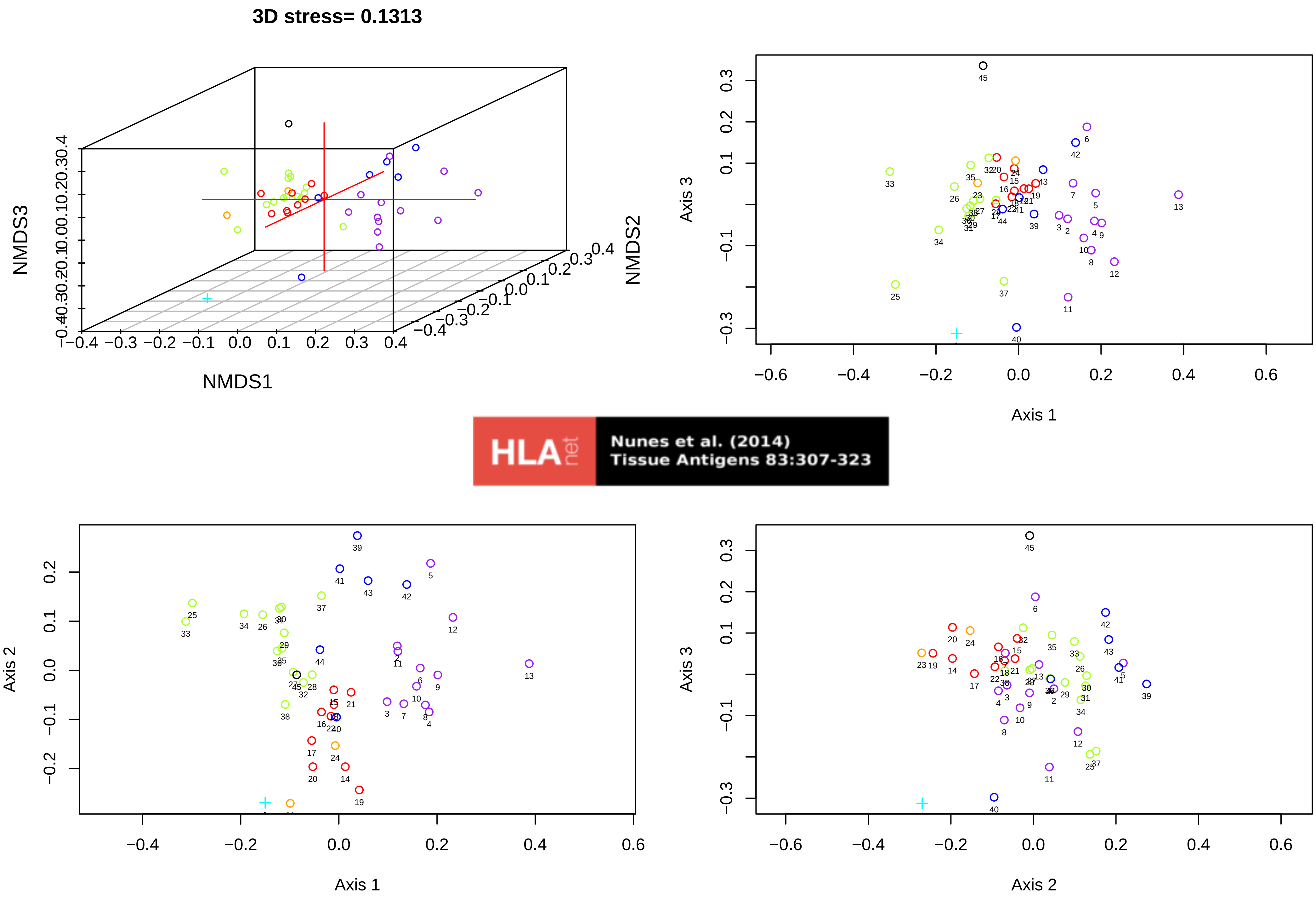
3D stress= 0.1313
NMDS1
NMDS2
NMDS3
Axis 1
Axis 3
HLA net
Nunes et al. (2014)
Tissue Antigens 83:307-323
Axis 1
Axis 2
Axis 2
Axis 3
